# Understanding Heterogeneous Drug Procurement Behaviour of Healthcare Institutions Under Pooled Procurement: Evidence From China

**DOI:** 10.34172/ijhpm.9372

**Published:** 2026-06-07

**Authors:** Boya Zhao, Zhao Cheng, Xing Lin Feng

**Affiliations:** School of Public Health, Peking University, Beijing, China.

**Keywords:** Pooled Procurement, Drug, Procurement Volume, Healthcare Institutions, Heterogeneity, China

## Abstract

**Background:**

While the overall impact of pooled drug procurement on drug procurement has been evaluated, little is known about how healthcare institutions’ pre-policy procurement composition is associated with heterogeneous post-policy procurement responses. This study addresses this gap under China’s National Volume-Based Procurement (NVBP) policy.

**Methods:**

We used monthly procurement data from January 2018 to December 2020 from four provinces in China, covering both pre- and post-policy periods, as the NVBP policy was implemented in January 2020. Outcomes were procurement volumes, measured as the number of defined daily doses (DDDs), of NVBP-covered drugs (bid-winning and non-winning products) and their clinically substitutable alternatives. All healthcare institutions in the selected provinces were included and stratified by (1) their pre-policy share of bid-winning products among NVBP drugs and (2) their pre-policy share of NVBP drugs among both NVBP and alternative drugs, to assess institutional heterogeneity. Interrupted time series (ITS) analysis was conducted to assess the immediate (level) and long-term (slope) post-policy changes.

**Results:**

Procurement volumes of bid-winning products increased substantially following the policy (level change: 1275%, *P*<.01), with smaller increases observed among healthcare institutions with higher pre-policy bid-winning shares (eg, highest-share subgroup: 441%, *P*<.01), consistent with uniform procurement targets. Although overall procurement of alternative drugs remained stable, healthcare institutions with higher pre-policy NVBP shares experienced significant increases, with level changes of up to 1010.9%, suggesting substitution driven by financial losses. In contrast, healthcare institutions with lower NVBP shares saw notable declines (level change: −34.8% to −65.2%), possibly reflecting demand-side substitution toward lower-priced NVBP drugs.

**Conclusion:**

Findings highlight that in pooled procurement, procurement behaviour is shaped more by regulatory enforcement and financial incentives than by price-driven demand. Strengthening guidance and oversight of supplier behaviour remains essential to realizing the full benefits of pooled procurement reforms.

## Background

**Key Messages**

**Implications for policy makers**

- Healthcare institutions’ pre-policy procurement pattern is associated with heterogeneous procurement responses to pooled drug procurement, suggesting that implementation effects are not uniform.
- Limiting pooled procurement to selected drugs may unintentionally encourage substitution toward alternative drugs; expanding coverage within therapeutic classes may mitigate this risk.
- Pre-policy procurement data can be used to identify healthcare institutions more likely to adjust behaviour after policy implementation and to guide risk-based monitoring.
- Healthcare institutions highly reliant on policy-covered drugs may shift toward alternatives to mitigate financial losses. Insights into procurement responses can guide complementary payment, performance evaluation, and compensation reforms to strengthen policy coherence and sustainability.

**Implications for the public**

This study helps the public understand how pooled drug procurement influences the drugs that hospitals choose to purchase under China’s National Volume-Based Procurement (NVBP) policy. While the policy has been effective in increasing the use of lower-priced, bid-winning drugs, it also shows that some hospitals, particularly those facing greater financial pressure after the policy, may increase their use of clinically substitutable drugs that are not covered by NVBP and are often more expensive. This shift may reduce the potential benefits of the policy for patients, including access to the most cost-effective treatments. To ensure that patients fully benefit from lower drug prices, effective government oversight and targeted regulatory strategies are essential. Using pre-policy procurement patterns to identify healthcare institutions at higher risk of unintended substitution and applying focused monitoring accordingly may help ensure that pooled procurement translates into real and sustained benefits for patients.

High pharmaceutical expenditures challenge the sustainability of health systems worldwide. In 2019, pharmaceutical spending averaged 16.2% of total health expenditure across the Organisation for Economic Co-operation and Development countries (6.6% in Denmark to 34.3% in Bulgaria).^1^ In China, the world’s second-largest economy, the share was higher, reaching 27.5% for inpatient and 40.6% for outpatient services.^2^ To alleviate financial pressures on both health plans and patients, various pharmaceutical pricing policies had been adopted to lower drug prices.^3^ Among them, pooled drug procurement has been implemented across diverse settings and proven effective.^3-13^ Its core mechanism lies in leveraging economies of scale and scope by consolidating purchasing power across institutions or jurisdictions, thereby enhancing bargaining capacity and reducing procurement costs.^3^

 However, the relationship between price reductions and total pharmaceutical spending is complicated by provider behavioural responses. Theoretical models of provider behaviour suggest that lower prices reduce the marginal benefit of dispensing affected drugs or services.^14^ To compensate for revenue losses, providers may increase the volume of low-priced items (the *income effect*) or shift toward non-regulated, higher-margin alternatives (the *substitution effect*). These patterns have been empirically observed in various contexts, including Medicare fee reductions in the United States, general practitioner service price regulation in France, and China’s zero mark-up drug policy.^15-22^ In the setting of pooled procurement, studies from China have also identified signs of substitution effects, reflected in increased procurement of non-covered drugs.^12,23,24^ However, these studies have largely focused on early pilot cities, limiting the generalizability of their findings.

 Importantly, theoretical models also suggest that the magnitude of income and substitution effects depends on the extent of revenue loss.^14^ Providers facing larger revenue reductions are more likely to exhibit stronger behavioural responses. While this relationship has been supported in other policy contexts, for example, hospitals in China that relied more heavily on drug revenues before the zero mark-up drug policy subsequently showed greater increases in diagnostic tests and medical consumables,^19^ its applicability to pooled procurement remains underexplored. Given the growing global adoption of pooled drug procurement, particularly in resource-constrained health systems, understanding these behavioural mechanisms is critical for designing effective cost-containment and regulatory strategies.

 China provides a particularly useful setting for such an investigation. Since late 2018, the National Healthcare Security Administration (NHSA) has implemented the National Volume-Based Procurement (NVBP), a nationwide pooled procurement initiative targeting widely used non-innovative drugs. Notably, the design of NVBP reveals a clear concern with potential provider responses. In China, public healthcare institutions are allowed to both prescribe and dispense drugs. Although they are prohibited from selling chemical drugs at a mark-up since 2012, informal payments or hidden rebates from manufacturers have been reported and remain a concern.^25-27^ NVBP has sought to eliminate such incentives by streamlining drug distribution and increasing procurement transparency for bid-winning products,^9^ theoretically reducing providers’ motivation to prescribe them. At the same time, drugs not covered by NVBP may still offer opportunities for financial gain through less regulated channels. To counterbalance this, NVBP introduced contractual requirements mandating that public institutions purchase 50%–70% of the previous year’s procurement volume for each NVBP-covered drug from the bid-winning suppliers. Additionally, the NHSA issued lists of therapeutic alternative drugs and instructed local authorities to closely monitor their procurement to prevent inappropriate substitution.

 Against this backdrop, we propose two hypotheses grounded in economic theory and policy design. First, we hypothesize that the procurement volume of bid-winning products increased following the implementation of NVBP, but the increase was smaller among institutions that had already procured a larger proportion of bid-winning products prior to the policy, as they were closer to fulfilling the mandated purchase targets (Hypothesis 1). Second, we hypothesize that the procurement of alternative drugs also increased after NVBP implementation, particularly among institutions that had previously procured more NVBP-covered medicines, as these institutions may have experienced greater revenue losses and thus were more inclined to substitute toward non-regulated alternatives (Hypothesis 2).

 This study draws on monthly procurement records from public healthcare institutions across several Chinese provinces to examine changes in the procurement volumes of NVBP-covered and alternative drugs before and after NVBP implementation. Furthermore, by leveraging variation in institutions’ baseline procurement patterns, we empirically test these two hypotheses. The findings offer valuable insights into the behavioural mechanisms shaping provider responses to price-focused reforms and can inform the development of targeted regulatory strategies to complement pooled procurement efforts in achieving more sustainable pharmaceutical spending.

## Methods

###  Data Sources and Sample Selection 

 We used monthly drug procurement records from healthcare institutions in four provinces in China. The data were obtained from the provincial Drug Tendering and Procurement System and include detailed information on drug generic name, formulation, specification, package size, manufacturer, procurement unit, unit price, procurement volume, procurement expenditure, procurement date (month-year), and healthcare institution ID, with no missing values for the variables used in the analysis. Prior to analysis, standard data cleaning procedures were applied, including internal consistency checks across procurement volume, unit price, and expenditure. No additional outlier exclusion or winsorization was performed.

 The four provinces are located in the eastern, southeastern, western, and northeastern regions of China, respectively. They were purposively selected from distinct geographic areas to enhance the representativeness of the study by encompassing heterogeneous economic conditions and healthcare system contexts across the country. In particular, eastern and southern regions generally have higher per capita gross domestic product, a higher density of tertiary healthcare institutions, and higher per capita healthcare expenditure than western and northern regions. All healthcare institutions within each selected province, including primary, secondary, and tertiary healthcare institutions, were included in the analysis; details on institutional characteristics are provided in Table. The study period spans from January 2018 to December 2020. All four provinces implemented the first round of the NVBP policy in January 2020.

**Table:** Characteristics of the Study Samples

**Characteristics**	**Number (%)**
Characteristics of healthcare institutions	7012
Locations	
Eastern province	2227 (31.8)
Southeastern province	2242 (32.0)
Western province	440 (6.3)
Northeastern province	2103 (30.0)
Levels	
Primary institutions	5716 (81.5)
Secondary institutions	939 (13.4)
Tertiary institutions	357 (5.1)
Characteristics of drug samples	
Number of NVBP-covered drugs	25
Anatomical main groups of NVBP-covered drugs	
Cardiovascular system	9
Nervous system	6
Anti-infective for systemic use	3
Anti-neoplastic and immunomodulating agents	3
Alimentary tract and metabolism	1
Blood and blood-forming organs	1
Musculo-skeletal system	1
Respiratory system	1
Number of bid-winning products	71
Number of non-winning products	303
Number of alternative drugs	71

Abbreviation: NVBP, National Volume-Based Procurement. Note: NVBP-covered and alternative drugs were identified based on the International Non-proprietary Names (INN) and administration routes. Specific products were identified by combining INN, manufacturer, and strength specifications.

 The study samples consist of NVBP-covered drugs in the first round of the NVBP policy and their officially certified alternatives. Both NVBP-covered and alternative drugs were identified based on the International Non-proprietary Names (INN) and administration routes. Specific products were identified by combining INN, manufacturer, and strength specifications. NVBP-covered drugs were further categorized into bid-winning and non-winning products using manufacturer information from the official bid results of the first NVBP round. Alternative drugs refer to drugs explicitly listed in official NHSA documents as clinically substitutable for NVBP-covered drugs, based on expert consensus from relevant clinical and pharmaceutical fields (eg, simvastatin as an alternative to atorvastatin; Table S1). To isolate the policy effect on NVBP drugs from that on their alternatives, we excluded alternative drugs that were covered by the first three rounds of NVBP policies during the study period. In total, we identify 25 NVBP drugs and 71 alternative drugs (See Supplementary file 1, Table S1). The final dataset comprises 128 130 monthly procurement records.

###  Measurement

####  Outcomes

 The primary outcome of interest was the procurement volume of the sample drugs, measured as *the number of Defined Daily Doses* (*DDDs*) procured by healthcare institutions each month. The *DDD* is a standardized unit established by the World Health Organization (WHO), representing the assumed average maintenance dose per day for a drug used for its main indication in adults. This measure allows for comparisons across drugs with different dosage forms and strengths. A higher number of DDDs generally reflects a greater quantity of drug procured. DDD values were obtained from the WHO Anatomical Therapeutic Chemical (ATC)/DDD Index, and for drugs without available DDD values, DDDs were calculated based on the WHO definition by referencing the corresponding drug package inserts. The secondary outcome was procurement expenditures, defined as the total monetary value of procured sample drugs each month and reported in Chinese yuan.

####  Baseline Variables

 To examine whether the policy effects on outcome variables varied by healthcare institutions’ baseline procurement patterns, we constructed two institution-level variables: the pre-policy share of bid-winning products (hereafter *bid-winning share*) and the pre-policy share of NVBP-covered drugs (hereafter *NVBP share*).

 The *bid-winning share* measures the extent to which a healthcare institution relied on bid-winning products within NVBP-covered drugs prior to policy implementation. It is defined as the average monthly proportion of procurement volume for bid-winning products relative to the total volume of NVBP-covered drugs during the pre-policy period (January 2018 to December 2019). The* NVBP share* captures the degree to which an institution relied on NVBP-covered drugs relative to all relevant options before the policy. It is defined as the average monthly proportion of procurement volume for NVBP-covered drugs relative to the total procurement volume of NVBP-covered and alternative drugs over the same pre-policy period. We used volume instead of expenditure to construct these two explanatory variables, as healthcare institutions in China plan procurement by volume, which better reflects institutional preferences and is less influenced by price variation.

###  Statistical Analyses

 This study used an interrupted time series (ITS) approach to evaluate changes in outcome variables before and after policy implementation. The implementation of the policy in January 2020 is treated as the intervention point. The period from January 2018 to December 2019 (24 months) is defined as the pre-policy period, while the period from January 2020 to December 2020 (12 months) is defined as the post-policy period. The model is specified as follows:


(1) $$Y_{t}=\beta_{0}+\beta_{1}T_{t}+\beta_{2}X_{t}+\beta_{3}X_{t}T_{t}+\varepsilon_{t}$$

In the model, *Y*_t_ denotes the outcome variable at time *t*, expressed in natural logarithms to address skewness and to allow regression coefficients to be interpreted as relative (percentage) changes, calculated as e^β^-1. *T*_t_ represents the time trend variable, typically specified as a linear function of time (eg, *t* = 1, 2, 3, …), and is used to capture the underlying secular trend in the outcomes. *X*_t_ is the intervention indicator, which takes the value of 1 if *t* falls within the post-policy period and 0 otherwise. *X*_t_*T*_t_ is the interaction term between the intervention indicator and the time trend, reflecting the direction and magnitude of changes in the time trend following the intervention. ε_t_ denotes the error term. β_0_ is the intercept, representing the average level of the outcome variable at the baseline, ie, prior to the policy. β_1 _captures the trajectory of the outcome variable prior to the policy. β_2 _measures the immediate level change in the outcome following policy implementation. β_3 _indicates the change in the slope of the outcome trend after the policy. To ensure the validity of statistical inference, serial autocorrelation in the regression residuals was assessed using the Cumby-Huizinga test. All regression models were estimated with Newey-West heteroskedasticity- and autocorrelation-consistent standard errors.^28^

 To examine heterogeneity in policy effects by institutions’ baseline procurement patterns, we conducted subgroup analyses by stratifying the sample into five subgroups based on pre-policy bid-winning share or pre-policy NVBP share. Because there are no established clinical or policy thresholds for these baseline procurement ratios, we used data-driven stratifications (eg, quintiles or fixed cutoffs) to flexibly capture nonlinear heterogeneity. For the pre-policy bid-winning share, which exhibited a right-skewed distribution, institutions were divided by quintiles into “very low,” “low,” “moderate,” “high,” and “very high” subgroups. Sensitivity analyses applied alternative stratifications based on the mean, median, and terciles. Given the substitution relationship between bid-winning and non-winning NVBP products, this subgroup/sensitivity analyses mainly focus on the procurement of bid-winning products, which implicitly reflects corresponding heterogeneity in institutional responses regarding non-winning products. For the pre-policy NVBP share, which was approximately normally distributed, five subgroups were similarly defined using fixed cutoffs at 0.2, 0.4, 0.6, and 0.8. Additional robustness checks used stratifications based on the mean, median, and a three-level classification (low: <0.33; moderate: 0.33-0.66; high: >0.66). To more precisely identify institutions likely to increase procurement of alternative drugs post-policy, we further subdivided the five fixed-cutoff subgroups into 0.05 and 0.01 increments to explore potential thresholds of pre-policy NVBP share and to provide a quantitative reference for targeted oversight.

 Furthermore, considering that the NHSA classifies alternative drugs in official documents into three categories based on descending clinical substitutability (completely, largely, and partially substitutable), we examined how healthcare institutions’ pre-policy NVBP share influenced their post-policy procurement behaviour separately for each of these three substitutability categories.

 To assess the robustness of our findings and further validate the estimated policy effects, we conducted a series of placebo tests. Given the absence of an external control group, we assigned fictitious policy intervention dates prior to the actual implementation of the NVBP (January 2020).^28^ Specifically, we re-estimated the ITS models assuming placebo intervention points set at June 2018, September 2018, December 2018, March 2019, and June 2019, corresponding to 6, 9, 12, 15, and 18 months before the actual intervention date (January 2020), respectively.

 STATA 17.0 was used for data analysis. The significance level was set as two-sided α < 0.05.

## Results

###  Samples and Characteristics

 A total of 7012 healthcare institutions across four Chinese provinces were included in the analyses, with most located in the eastern (31.8%), southeastern (32.0%), and northeastern (30.0%) regions, and fewer in the western region (6.3%), reflecting regional disparities in health resource distribution. Primary care facilities comprised the majority (81.5%), followed by secondary (13.4%) and tertiary (5.1%) institutions. These institutions generated 128 130 procurement records during the study period, involving 25 NVBP-covered and 71 alternative drugs (Table, Table S1). These NVBP-covered drugs were classified into eight anatomical main groups based on the ATC classifications, with cardiovascular system drugs accounting for the largest number (n = 9). According to the bidding results in the first round of NVBP, for the NVBP-covered drugs, 71 bid-winning and 303 non-winning products were purchased during the study period.

###  Overall Changes in Procurement Volumes

Figure 1 and Table S2 present the results of the ITS analyses for the procurement volume of NVBP-covered and alternative drugs, and results for procurement expenditures are listed in Table S3. Prior to the NVBP policy, there were no clear trends or only modest upward trends in procurement volumes for these drugs. Following the policy implementation, the procurement volume of bid-winning products rose sharply by approximately 1275% (β_2_ = 2.606, *P* < .01), while that of non-winning products significantly dropped by about 69.5% (β_2_ = -1.185, *P* < .01). No significant post-policy trend changes were observed for either bid-winning or non-winning drugs. Procurement volumes of alternative drugs remained unchanged after the policy. However, procurement expenditures for bid-winning products increased only modestly, suggesting a combined effect of price reductions and volume growth.

**Figure 1:**
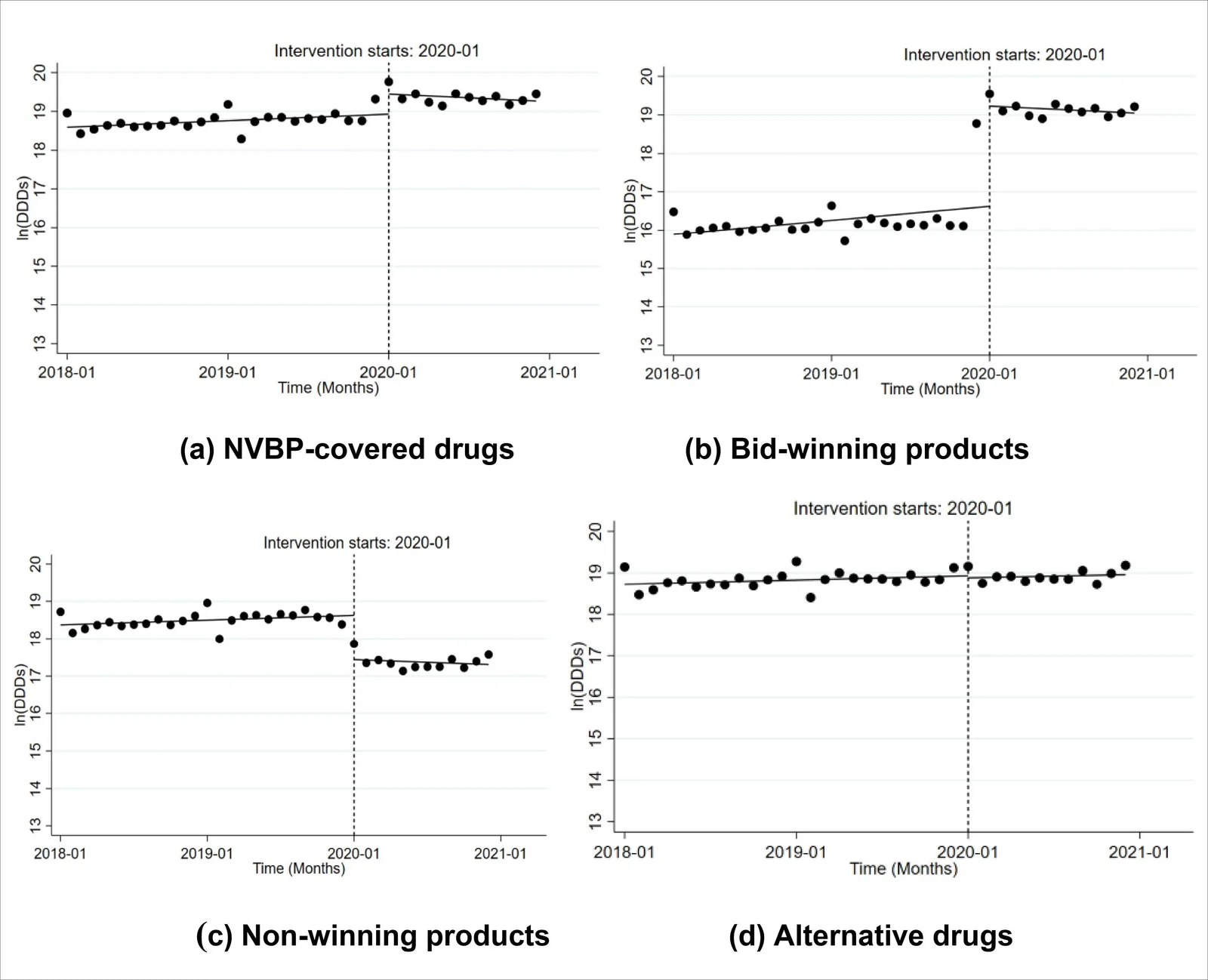
Estimated Trends of Procurement Volumes From Interrupted Time Series in the Main Analysis. Abbreviations: NVBP, National Volume-Based Procurement; DDDs, defined daily doses.

###  Heterogeneity in Policy Responses by Pre-Policy Bid-Winning Share

 To examine whether the policy effects on the procurement volume of bid-winning products varied by institutions’ baseline procurement patterns of bid-winning versus non-winning products, we first described the distribution of the pre-policy bid-winning share. As shown in Figure 2a, the distribution is right-skewed, with approximately 95% of institutions having a pre-policy bid-winning share below 0.5, indicating that most institutions procured more non-winning than bid-winning products before the policy. Table S4 presents the number of institutions and the share values in each subgroup with a difference in pre-policy bid-winning share. In the five-level stratification based on quintiles, the average bid-winning shares from highest to lowest were 0.43, 0.19, 0.12, 0.08, and 0.04.

**Figure 2:**
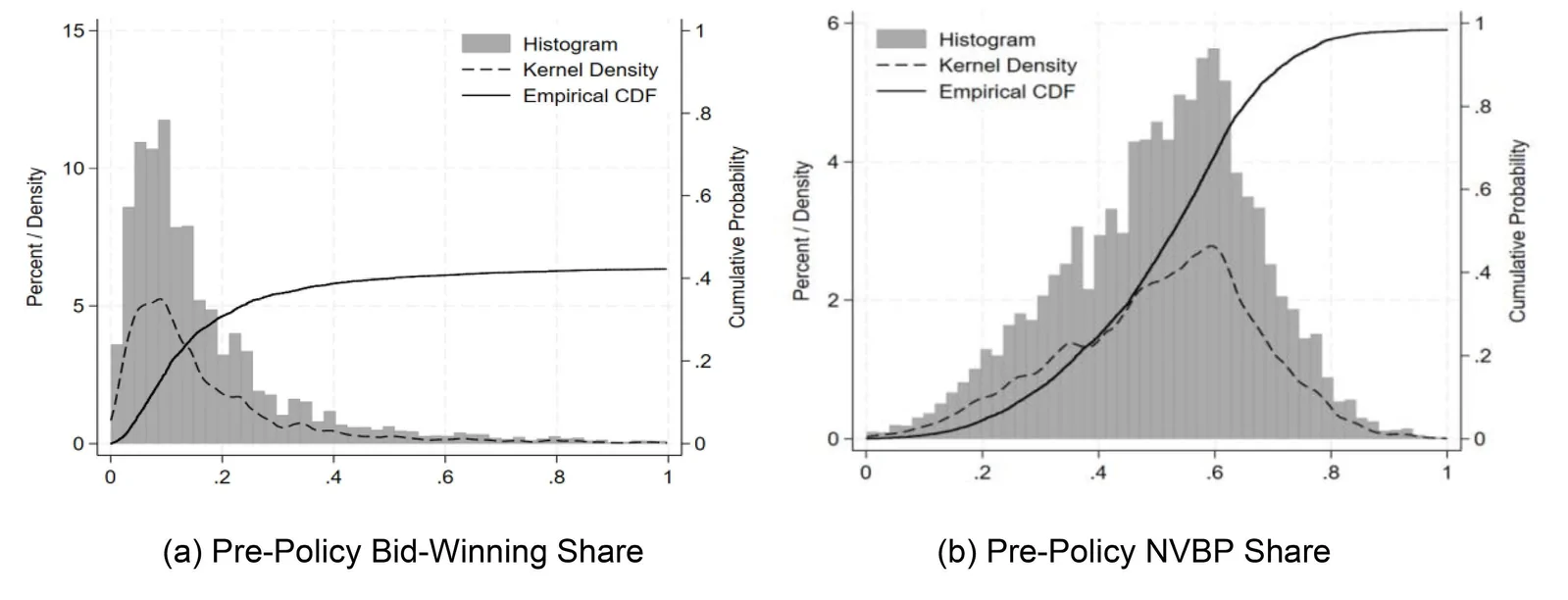
Distribution for Two Explanatory Variables. Abbreviations: NVBP, National Volume-Based Procurement; CDF, cumulative distribution function.

 Results from the subgroup ITS regressions on procurement volume and expenditure of bid-winning products are reported in Tables S5-S6 and Figure S1 (See Supplementary files 1 and 2). Figure 3 also summarizes the post-policy level changes (β₂) in volume from the quintile-based subgroup analysis in a forest plot; trend changes (β₃) are not shown, as they were consistently insignificant. The estimated level changes were all significantly positive but declined as the pre-policy bid-winning share increased. For instance, in the very low share subgroup, the procurement volume of bid-winning products increased by 3623% (β₂ = 3.617, *P* < .01) after the policy, compared to 441% (β₂ = 1.688, *P* < .01) in the very high share subgroup.

**Figure 3:**
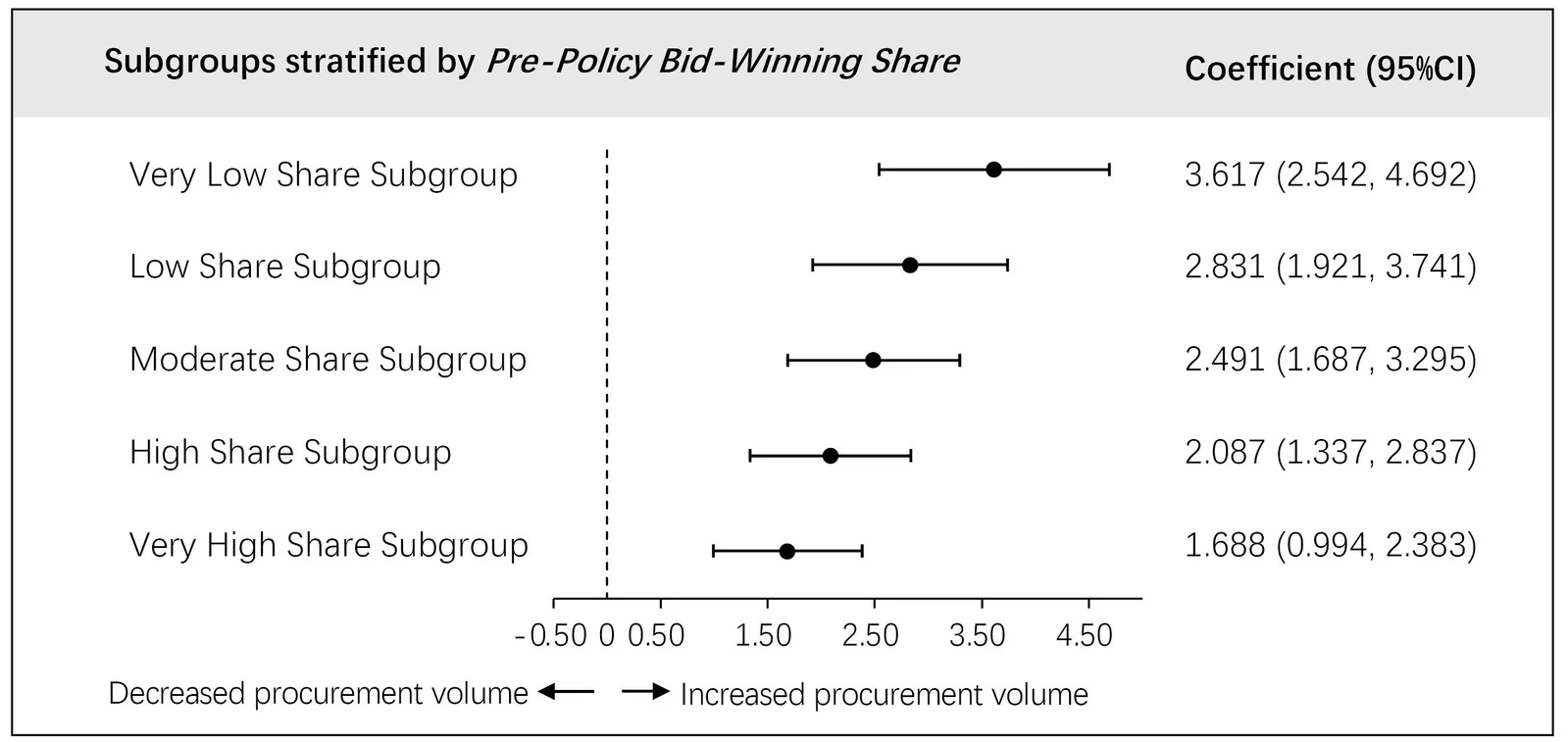
ITS-Based Forest Plot of Post-Policy Changes in Bid-Winning Product Procurement Volume by Pre-Policy Bid-Winning Share (Five-Level Stratification). Abbreviations: CI, confidence interval; ITS, interrupted time series. Note: The figure only displays estimated post-policy level changes (β₂) and their 95% CIs from ITS regressions. Trend changes (β₃) are not shown, as they were consistently insignificant. For all regression results, please refer to Table S5 in Supplementary file 1.

 We also calculated the post-policy bid-winning share for each subgroup, which hovered around 85%, as shown in Table S7. Specifically, under the five-level stratification, the post-policy shares from the very low to very high subgroups were 76.1%, 80.9%, 80.9%, 79.5%, and 76.1%, respectively. The subgroup analyses based on mean, median, and tertile splits yielded consistent trends, reinforcing the robustness of the quintile-based results (Tables S8–S10, Figure S2-S4).

###  Heterogeneity in Policy Responses by Pre-Policy NVBP Share

 To explore how institutions’ baseline procurement patterns influenced their post-policy purchasing behaviour regarding alternative drugs, we examined heterogeneity in response by stratifying institutions based on their pre-policy NVBP share. Figure 2b shows that the distribution of this share is approximately normal, with a slight left skew. Notably, about 60% of institutions exhibited a value above 0.5, indicating a stronger procurement preference for NVBP-covered drugs relative to their alternatives. The remaining 40% relied more on alternative drugs before the policy. Table S11 summarizes the sample sizes and average share values for each subgroup. Under the five-level classification, the mean NVBP shares from highest to lowest were 0.85, 0.67, 0.51, 0.31, and 0.14.

 ITS regression results by subgroup are reported in Tables S12-S13 and Figure S5 (Supplementary files 2 and 3), and estimated post-policy level changes (β₂) from the five-level stratification analysis are visualized in Figure 4. The effects reveal a clear reversal across the NVBP share distribution: institutions with lower baseline shares experienced marked declines in the procurement of alternative drugs, whereas those with higher shares exhibited substantial increases. The procurement volume of alternative drugs fell by 65.2% (β₂ = –1.055, *P* < .01) and 34.8% (β₂ = –0.428, *P* < .01) in the very low and low share subgroups, respectively. Conversely, in the high and very high share subgroup, procurement rose significantly by 18.1% (β₂ = 0.166, *P* < .05) and 1010.9% (β₂ = 2.405, *P* < .01) following policy implementation. These findings align with the expectation that providers more reliant on NVBP-covered drugs prior to the policy—and thus more affected by associated revenue reductions—were more likely to shift toward non-regulated alternatives with higher potential margins. For the moderate share subgroup (pre-policy NVBP share: 0.4-0.6), there was no significant change in the procurement of alternative drugs. By further subdividing this subgroup in 0.05 and 0.01 increments, we identified 0.42 as the threshold of pre-policy NVBP share above which healthcare institutions increased procurement of alternative drugs post-policy (Figure S6).

**Figure 4:**
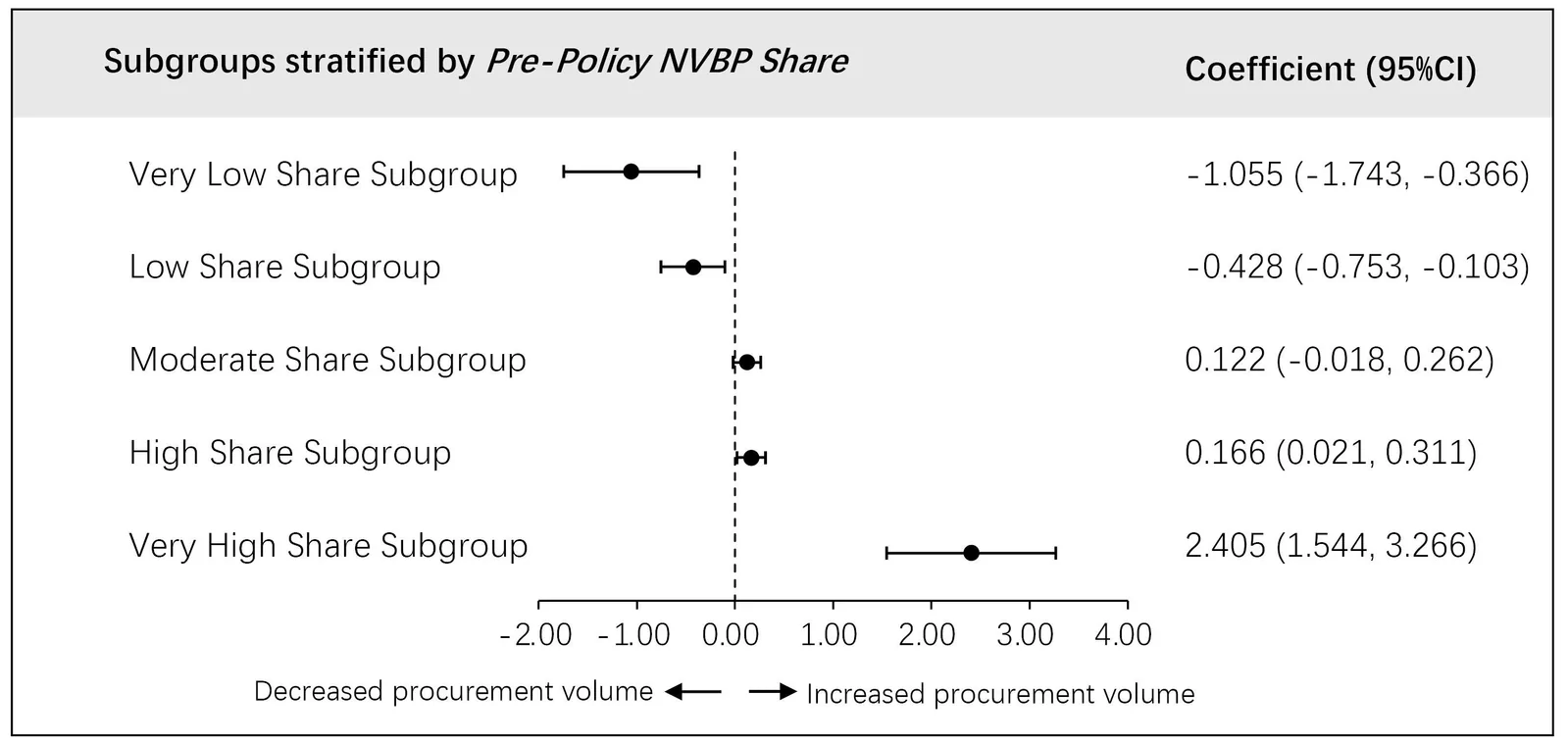
ITS-Based Forest Plot of Post-Policy Changes in Alternative Drug Procurement Volume by Pre-Policy NVBP Share (Five-Level Stratification). Abbreviations: NVBP, National Volume-Based Procurement; CI, confidence interval; ITS, interrupted time series. Note: The figure only displays estimated post-policy level changes (β₂) and their 95% confidence intervals from ITS regressions. Trend changes (β₃) are not shown, as they were consistently insignificant. For all regression results, please refer to Table S12 in Supplementary file 3.

 Similar patterns were observed in the two-level and three-level stratification subgroup analyses, further supporting the robustness of the findings (Tables S14–S16, Figure S7-S9). Results for the three alternative drug categories by substitutability are presented in Figure S10. For each substitutability category, we observed that as the pre-policy NVBP share increased, post-policy procurement of alternative drugs initially decreased and then increased, which are consistent with the above analysis.

 The results of these placebo tests are presented in Tables S17-S20. For each of these placebo dates, neither the estimated immediate effect nor the long-term change was statistically significant. These findings suggest that the observed changes in the outcomes can be confidently attributed to the NVBP policy implementation, rather than pre-existing trends or other confounding factors.

## Discussion

###  Main Finding

 This study examined provider responses to pooled procurement in the context of China’s NVBP policy, using monthly procurement data from public healthcare institutions across multiple provinces. Overall, we found that the procurement volume of bid-winning products increased significantly after the policy, while the volume of alternative drugs remained largely unchanged. However, subgroup analyses revealed patterns that diverged from the aggregate trends: institutions with lower pre-policy bid-winning shares showed greater increases in bid-winning product procurement, while those with higher pre-policy NVBP shares experienced notable increases in alternative drug procurement. These findings suggest that providers adjusted their behaviour in line with procurement obligations and potential financial incentives, reflecting a consistent and strategic response to the policy.

###  Interpretation

 We observed a significant increase in the procurement volume of bid-winning products and a concurrent decline in non-winning products following NVBP implementation, which supports our first hypothesis and aligns with previous studies.^12,29^ This pattern is consistent with the policy’s design, which requires healthcare institutions to procure a minimum mandated procurement ratio of NVBP-covered medicines from bid-winning manufacturers. As a result, institutions substituted non-winning products with their bid-winning counterparts to meet these requirements. Moreover, we found that institutions that had already procured a larger proportion of bid-winning products before the policy showed smaller increases in procurement volume of bid-winning products post-policy. This finding is intuitive. Since the mandated procurement ratio was uniformly applied, those with higher pre-policy shares were already close to meeting the requirement, leaving less room for further adjustment.

 Interestingly, the average post-policy bid-winning share reached approximately 80%, exceeding the mandated procurement ratio of 50%–70%. Given that bid-winning products no longer generate financial returns for providers under the NVBP policy, this overshooting cannot be simply interpreted as a supply-side income effect. Instead, it may plausibly reflect demand-side responses, as prior studies show that reductions in drug prices or patient cost-sharing can improve affordability and address unmet treatment needs, thereby increasing medication use. This finding highlights the importance of considering demand-side reactions when designing and implementing supply-side procurement policies. Alternatively, the observed overshooting may also reflect over-compliance during the early phase of policy implementation, as healthcare institutions may respond to regulatory uncertainty by exceeding mandated targets, consistent with transitional dynamics under newly introduced administrative procurement mechanisms.^30^ Whether procurement patterns stabilize at different levels over time warrants further investigation.^31-34^

 The observed heterogeneity in alternative drug procurement across institutions may reflect a combination of both supply-side and demand-side factors. Institutions with higher pre-policy NVBP shares showed notable increases in alternative drug purchases after the policy. On the supply side, this pattern may be related to greater financial pressure faced by these institutions under the NVBP scheme. In China, longstanding concerns exist about provider-industry relationships, with under-the-table commissions from pharmaceutical companies widely reported.^25-27^ By enhancing procurement transparency and centralized distribution, NVBP effectively eliminated informal financial returns from bid-winning products. In response, providers may have had incentives to shift toward alternative medicines that could potentially offer financial returns through non-transparent channels. Such behaviour runs counter to the cost-containment goals of pooled procurement. On the demand side, we cannot rule out the role of clinical or patient preferences in shaping the use of alternative drugs. In the first round of NVBP, most of the bid-winning products were generics. Some patients and physicians may remain hesitant to use generic bid-winning products due to perceived concerns about the efficacy or safety of generic drugs, as suggested in prior studies.^35,36^ As a result, they may shift toward brand-name alternative drugs. Brand-name non-winning products within NVBP-covered drugs are less likely to be chosen, as healthcare institutions are subject to regulatory assessments of procurement ratios between bid-winning and non-winning products within NVBP-covered drugs, which may discourage purchasing or prescribing non-winning products. Institutions with lower pre-policy NVBP shares exhibited reductions in alternative procurement. These providers may have faced relatively limited financial losses under the policy and therefore may have had fewer incentives to increase alternative use. Meanwhile, significant price reductions for bid-winning products may have spurred patient demand for lower-cost bid-winning products, potentially contributing to demand-side substitution away from alternatives.

###  Policy Implications

 This study has several policy implications. First, our findings confirm that substitution effects exist under pooled procurement. To mitigate this, pooled procurement schemes should ideally include all interchangeable drugs within a therapeutic class, thereby minimizing incentives for inappropriate substitution across generic molecules. Second, we find that both the likelihood and magnitude of substitution effects are associated with the potential financial losses faced by providers, measured by pre-policy NVBP share in this study. This suggests that policy-makers can use historical procurement data to identify high-risk institutions in advance and develop targeted oversight or preventive strategies. For instance, our findings indicate that healthcare institutions with a pre-policy NVBP share above 0.42 increased procurement of alternative drugs post-policy. Third, the underlying mechanism of substitution effects lies in the existence of financial incentives for dispensing alternative medicines. This implies that adopting zero markup drug policies may not be sufficient to break the economic link between providers and pharmaceutical manufacturers. Instead, combining zero markup drug reforms with pooled procurement may be a more effective approach to disrupting these incentives and reducing the risk of inappropriate drug use. Together, these insights underscore the importance of aligning policy design with provider incentives to ensure the long-term success of cost-containment efforts of pooled procurement.

###  Strengths and Limitations

 This study is the first to use cross-provincial data to examine how China’s NVBP policy affected public healthcare institutions’ procurement of NVBP-covered and alternative medicines, whereas most prior studies focused on single provinces/cities or specific therapeutic classes. Our findings also provide novel evidence that procurement behaviours varied by baseline procurement patterns under pooled procurement. These insights help clarify providers’ behavioural mechanisms and can inform policy-makers in designing targeted regulatory strategies for future pharmaceutical pricing reforms.

 This study has several limitations. First, the data cover only four provinces in China. Although these provinces span geographically and socioeconomically diverse regions, the sample is not statistically representative in a probabilistic sense, which may limit the generalizability of the findings. Second, although prior studies suggest that both supply-side and demand-side factors may influence drug procurement behaviour, the procurement data used in this study do not allow us to directly and empirically test or distinguish between these mechanisms, particularly the demand-side channels. Therefore, the causal interpretability of our findings is limited. Third, due to the absence of institution-level financial outcome data (eg, revenue or profit changes), we are unable to quantify the actual financial losses faced by healthcare institutions or to directly test incentive-driven substitution mechanisms. Interpretations regarding financial motivations should therefore be viewed as exploratory rather than causal. Fourth, our analysis focuses on short-term impacts within one year of policy implementation, which likely capture healthcare institutions’ initial responses to the administrative procurement mechanism. These short-term effects may not represent market equilibrium outcomes or be directly extrapolated to longer-term impacts, because provider behaviour may continue to evolve over time as institutions, clinicians, and patients adapt to the policy. Further research on longer-term effects is warranted. Finally, as NVBP is a nationwide policy, no control group was available, which limits our ability to draw strong causal inferences about the policy’s effects.

## Conclusions

 This study examined provider responses to China’s NVBP policy and found that while procurement of bid-winning drugs increased overall, the extent of change varied by baseline procurement patterns. Institutions more exposed to potential financial losses showed stronger substitution toward alternative drugs, suggesting the presence of supply-driven incentives. These findings indicate that provider behaviour under pooled procurement is shaped more by regulatory mandates and financial incentives than by demand-side price responsiveness. To maximize the intended benefits of such reforms, it is crucial to design pooled procurement policies that ensure comprehensive coverage within therapeutic classes and enable targeted oversight of high-risk institutions. Strengthening the alignment between policy design and provider incentives remains essential to reducing unintended behaviours and promoting more sustainable and rational medicine use.

## Disclosure of artificial intelligence (AI) use

 Grammarly (https://app.grammarly.com/) was employed to refine the grammar, spelling, and punctuation of this manuscript, which was subsequently reviewed and finalized by the authors.

## Ethical issues

 This study used de-identified administrative drug procurement data and did not involve human participants; therefore, ethics approval and informed consent were waived.

## Conflicts of interest

 Authors declare that they have no conflicts of interest.

## Disclaimer

 No proprietary or commercial interests were involved in the design, conduct, analysis, or reporting of this research.

## Data availability statement

 The data are not publicly available due to administrative restrictions, but may be accessed upon reasonable request and subject to approval by the data custodians.

## Supplementary files


Supplementary file 1 contains Tables S1-S10.

Supplementary file 2 contains Figures S1-S10.

Supplementary file 3 contains Tables S11-S20.
