# Supplementary material for: Understanding Heterogeneous Drug Procurement Behaviour of Healthcare Institutions Under Pooled Procurement: Evidence From China
Source: Int J Health Policy Manag. 2026 Jun 7;15:9372. doi: 10.34172/ijhpm.9372 (PMC13729802; doi:10.34172/ijhpm.9372)
Supplement: Supplementary file 1 [file ijhpm-15-9372-s001.pdf]

Article title: Understanding Heterogeneous Drug Procurement Behaviour of Healthcare Institutions Under Pooled Procurement: Evidence From China

Journal name: International Journal of Health Policy and Management (IJHPM)

Authors' information: Boya Zhao, Zhao Cheng, Xing Lin Feng*

School of Public Health, Peking University, Beijing, China.

***Correspondence to:** Xing Lin Feng; Email: fxl@bjmu.edu.cn

Citation: Zhao B, Cheng Z, Feng XL. Understanding heterogeneous drug procurement behaviour of healthcare institutions under pooled procurement: evidence from China. Int J Health Policy Manag. 2026;15:9372. doi:10.34172/ijhpm.9372

Supplementary file 1

List of contents

Table S1. International Nonproprietary Names of NVBP-Covered Medicines and Corresponding Alternative Medicines 2

Table S2. ITS Regression Results for Procurement Volume (DDDs) in the main analyses.....................4

Table S3. ITS Regression Results for Procurement Expenditures (CNY) in the main analyses 4

Table S4. Descriptive Statistics of Institutional Subgroups by Pre-Policy Bid-Winning Share 5

Table S5. ITS Regression Results for Procurement Volume of Bid-Winning Products by Pre-Policy Bid-Winning Share (Five-Level Stratification) 5

Table S6. ITS Regression Results for Procurement Expenditure of Bid-Winning Products by Pre-Policy Bid-Winning Share (Five-Level Stratification) 6

Table S7. Post-Policy Bid-winning Share for Institutional Subgroups Stratified by Pre-Policy Bid-Winning Share 6

Table S8. Sensitivity Analyses: ITS Regression Results on Bid-Winning Product Procurement by Pre-Policy Bid-Winning Share (Three-Level Stratification) 7

Table S9. Sensitivity Analyses: ITS Regression Results on Bid-Winning Product Procurement by Pre-Policy Bid-Winning Share (Median split) 8

Table S10. Sensitivity Analyses: ITS Regression Results on Bid-Winning Product Procurement by Pre-Policy Bid-Winning Share (Mean split)...8

Table S1. International Nonproprietary Names of NVBP-Covered Medicines and Corresponding Alternative Medicines

INN of NVBP-covered Drugs (25)	Alternative Drugs (71)
Atorvastatin (Oral)	Pravastatin, Fluvastatin, Simvastatin, Lovastatin, XueZhiKang, ZhiBiTai
Rosuvastatin (Oral)	Pravastatin, Fluvastatin, Simvastatin, Lovastatin, XueZhiKang, ZhiBiTai
Clopidogrel (Oral)	Aspirin, Cilostazol
Irbesartan (Oral)	Telmisartan, Azilsartan Medoxomil, Olmesartan Medoxomil/Amlodipine, Valsartan/Amlodipine, Olmesartan Medoxomil/Hydrochlorothiazide, Losartan Potassium/Hydrochlorothiazide, Telmisartan/Hydrochlorothiazide, Valsartan/Hydrochlorothiazide, Candesartan Cilexetil/Hydrochlorothiazide
Amlodipine (Oral)	Felodipine, Nifedipine, Amlodipine/Atorvastatin, Amlodipine/Benazepril, Olmesartan Medoxomil/Amlodipine, Valsartan/Amlodipine

Entecavir (Oral)	Tenofovir Alafenamide Fumarate, Lamivudine, Telbivudine
Escitalopram (Oral)	Fluvoxamine, Bupropion, Trazodone
Paroxetine (Oral)	Fluvoxamine, Bupropion, Trazodone
Olanzapine (Oral)	Paliperidone, Clozapine, Aripiprazole, Amisulpride, Loxapine Succinate, Haloperidol, Perphenazine Decanoate, Chlorpromazine, Ziprasidone
Cefuroxime (Oral)	Cefaclor, Cephalexin, Cefprozil, Cefotiam, Cefixime, Cefadroxil
Risperidone (Oral)	Aripiprazole, Amisulpride, Loxapine Succinate, Perphenazine, Haloperidol, Perphenazine Decanoate, Chlorprothixene, Paliperidone, Sulpiride, Fluphenazine, Chlorpromazine, Ziprasidone
Gefitinib (Oral)	Icotinib, Erlotinib, Afatinib, Osimertinib
Fosinopril (Oral)	Benazepril, Perindopril, Amlodipine/Benazepril, Enalapril/Folic acid, Perindopril/Indapamide
Irbesartan/Hydrochlorot hiazide (Oral)	Olmesartan Medoxomil/Hydrochlorothiazide, Losartan Potassium/Hydrochlorothiazide, Telmisartan/Hydrochlorothiazide, Valsartan/Hydrochlorothiazide, Candesartan Cilexetil/Hydrochlorothiazide, Telmisartan, Azilsartan Medoxomil, Olmesartan Medoxomil/Amlodipine, Valsartan/Amlodipine
Lisinopril (Oral)	Benazepril, Perindopril, Amlodipine/Benazepril, Enalapril/Folic acid, Perindopril/Indapamide
Tenofovir disoproxil (Oral)	Tenofovir Alafenamide Fumarate, Lamivudine, Telbivudine
Losartan (Oral)	Telmisartan, Azilsartan Medoxomil, Olmesartan Medoxomil/Amlodipine, Valsartan/Amlodipine, Olmesartan Medoxomil/Hydrochlorothiazide, Losartan Potassium/Hydrochlorothiazide, Candesartan Cilexetil/Hydrochlorothiazide
Enalapril (Oral)	Benazepril, Perindopril, Amlodipine/Benazepril, Enalapril/Folic acid, Perindopril/Indapamide
Levetiracetam (Oral)	Oxcarbazepine, Magnesium Valproate, Sodium Valproate, Carbamazepine, Lamotrigine, Topiramate
Imatinib (Oral)	Nilotinib, Dasatinib
Montelukast (Oral)	Pemirolast, Pranlukast, Cetirizine
Montmorillonite (Oral)	Loperamide, Tannalbin

Pemetrexed (Injectable)<br>Flurbiprofen axetil (Injectable)<br>Dexmedetomidine (Injectable)	Piroxicam, Diclofenac,<br>Ketorolac Tromethamine, Indomethacin

Note:
1. Alternative drugs that were listed as NVBP-covered drugs in the second or third round of the National Volume-Based Procurement(NVBP) were excluded to avoid potential confounding effects from overlapping policy interventions.
2. XueZhiKang, ZhiBiTuo and ZhiBiTai are Chinese traditional medicines.

Table S2. ITS Regression Results for Procurement Volume (DDDs) in the main analyses

	NVBP-covered medicines			Alternative medicines
	Bid-winning products	Non-winning products	Total	Total
Constant	15.89***	18.37***	18.59***	18.73***
	(0.204)	(0.0686)	(0.0798)	(0.0907)
T_t	0.0306	0.0105**	0.0140**	0.00867
	(0.0250)	(0.00505)	(0.00643)	(0.00606)
X_t	2.606***	-1.185***	0.517***	-0.0489
	(0.428)	(0.174)	(0.150)	(0.125)
X_tT_t	-0.0470	-0.0222	-0.0304*	-0.00177
	(0.0298)	(0.0202)	(0.0174)	(0.0176)

Note: Standard errors in parentheses. *, **, and *** indicate statistical significance at the 10%, 5%, and 1% levels, respectively. The same applies below.

Table S3. ITS Regression Results for Procurement Expenditures (CNY) in the main analyses

	NVBP-covered medicines			Alternative medicines
	Bid-winning products	Non-winning products	Total	Total
Constant	18.10***	20.14***	20.38***	19.46***
	(0.0876)	(0.0751)	(0.0682)	(0.0831)
T_t	0.0164**	0.0132**	0.0115**	0.0190***
	(0.00812)	(0.00555)	(0.00476)	(0.00553)
X_t	0.416*	-1.062***	-0.751***	-0.0224
	(0.215)	(0.211)	(0.189)	(0.125)
X_tT_t	-0.00767	-0.0585**	-0.0330	-0.0168
	(0.0251)	(0.0274)	(0.0249)	(0.0167)

Table S4. Descriptive Statistics of Institutional Subgroups by Pre-Policy Bid-Winning Share

Subgroup	Numbers of institutions	Pre-Policy Bid-Winning Share			
		Mean	Std. Dev.	Min	Max
**Binary Stratification (Median split)**					
Low Share Subgroup	27,071	0.0666	0.0297	0.0004	0.1166
High Share Subgroup	27,049	0.2729	0.1720	0.1169	0.9974
**Binary Stratification (Mean split)**					
Low Share Subgroup	36,025	0.0851	0.0418	0.0004	0.1696
High Share Subgroup	18,095	0.3382	0.1768	0.1702	0.9974
**Three-level Stratification**					
Low Share Subgroup	18,065	0.0500	0.0212	0.0004	0.0843
Moderate Share Subgroup	18,033	0.1206	0.0238	0.0843	0.1709
High Share Subgroup	18,022	0.3389	0.1768	0.1714	0.9974
**Five-level Stratification**					
Very Low Share Subgroup	10,832	0.0358	0.0146	0.0004	0.0582
Low Share Subgroup	10,847	0.0777	0.0112	0.0582	0.0976
Moderate Share Subgroup	10,795	0.1188	0.0142	0.0977	0.1449
High Share Subgroup	10,822	0.1882	0.0290	0.1449	0.2437
Very High Share Subgroup	10,824	0.4282	0.1782	0.2439	0.9974

Table S5. ITS Regression Results for Procurement Volume of Bid-Winning Products by Pre-Policy Bid-Winning Share (Five-Level Stratification)

	Very Low Share Subgroup	Low Share Subgroup	Moderate Share Subgroup	High Share Subgroup	Very High Share Subgroup
T_t	0.0391	0.0455*	0.0351	0.0163	0.0179
	(0.0324)	(0.0272)	(0.0242)	(0.0221)	(0.0203)
X_t	3.617***	2.831***	2.491***	2.087***	1.688***
	(0.548)	(0.465)	(0.410)	(0.382)	(0.354)

X_tT_t	-0.0627*	-0.0775**	-0.0656**	-0.0373	-0.0360
	(0.0360)	(0.0325)	(0.0291)	(0.0271)	(0.0266)
Constant	13.30***	14.13***	14.49***	14.71***	14.35***
	(0.259)	(0.224)	(0.203)	(0.178)	(0.172)

Table S6. ITS Regression Results for Procurement Expenditure of Bid-Winning Products by Pre-Policy Bid-Winning Share (Five-Level Stratification)

	Very Low Share Subgroup	Low Share Subgroup	Moderate Share Subgroup	High Share Subgroup	Very High Share Subgroup
T_t	0.0109	0.0183**	0.0268***	0.0136**	0.0107*
	(0.00949)	(0.00922)	(0.00913)	(0.00545)	(0.00597)
X_t	0.843***	0.645***	0.401*	0.0373	-0.0844
	(0.248)	(0.224)	(0.216)	(0.119)	(0.202)
X_tT_t	-0.0155	-0.0314	-0.0359	-0.00758	-0.00288
	(0.0271)	(0.0257)	(0.0249)	(0.0195)	(0.0242)
Constant	16.36***	16.54***	16.34***	16.63***	16.54***
	(0.0860)	(0.0936)	(0.106)	(0.0647)	(0.0801)

Table S7. Post-Policy Bid-winning Share for Institutional Subgroups Stratified by Pre-Policy Bid-Winning Share

**Subgroup**	**Before the policy implementation**	**After the policy implementation**
**Binary Stratification (Median split)**		
Low Share Subgroup	6.66%	79.06%

High Share Subgroup	27.29%	76.95%
**Binary Stratification (Mean split)**		
Low Share Subgroup	8.51%	79.63%
High Share Subgroup	33.82%	75.34%
**Three-level Stratification**		
Low Share Subgroup	5.00%	78.29%
Moderate Share Subgroup	12.06%	80.94%
High Share Subgroup	33.89%	76.89%
**Five-level Stratification**		
Very Low Share Subgroup	3.58%	76.07%
Low Share Subgroup	7.77%	80.88%
Moderate Share Subgroup	11.88%	80.94%
High Share Subgroup	18.82%	79.49%
Very High Share Subgroup	42.82%	76.12%

Table S8. Sensitivity Analyses: ITS Regression Results on Bid-Winning Product Procurement by Pre-Policy Bid-Winning Share (Three-Level Stratification)

	Low Share Subgroup	Moderate Share Subgroup	High Share Subgroup
T_t	0.0435<br>(0.0299)	0.0335<br>(0.0245)	0.0171<br>(0.0208)
X_t	3.284^{***}	2.493^{***}	1.830^{***}

	(0.507)	(0.419)	(0.362)
X_tT_t	-0.0737**	-0.0594**	-0.0389
	(0.0339)	(0.0297)	(0.0267)
Constant	14.10***	15.04***	15.01***
	(0.245)	(0.201)	(0.172)

Table S9. Sensitivity Analyses: ITS Regression Results on Bid-Winning Product Procurement by Pre-Policy Bid-Winning Share (Median split)

	Low Share Subgroup	High Share Subgroup
T_t	0.0430	0.0205
	(0.0281)	(0.0217)
X_t	2.980***	2.118***
	(0.476)	(0.376)
X_tT_t	-0.0709**	-0.0162
	(0.0325)	(0.0271)
Constant	14.83***	15.48***
	(0.230)	(0.178)

Table S10. Sensitivity Analyses: ITS Regression Results on Bid-Winning Product Procurement by Pre-Policy Bid-Winning Share (Mean split)

	Low Reliance Group	High Reliance Group
T_t	0.0370	0.0171
	(0.0265)	(0.0208)
X_t	2.823***	1.861***
	(0.452)	(0.364)
X_tT_t	-0.0651**	0.0174
	(0.0311)	(0.0267)
Constant	15.37***	15.01***
	(0.217)	(0.172)
