# Supplementary material for: Understanding Heterogeneous Drug Procurement Behaviour of Healthcare Institutions Under Pooled Procurement: Evidence From China
Source: Int J Health Policy Manag. 2026 Jun 7;15:9372. doi: 10.34172/ijhpm.9372 (PMC13729802; doi:10.34172/ijhpm.9372)
Supplement: Supplementary file 2 [file ijhpm-15-9372-s002.pdf]

Article title: Understanding Heterogeneous Drug Procurement Behaviour of Healthcare Institutions Under Pooled Procurement: Evidence From China

Journal name: International Journal of Health Policy and Management (IJHPM)

Authors' information: Boya Zhao, Zhao Cheng, Xing Lin Feng*

School of Public Health, Peking University, Beijing, China.

***Correspondence to:** Xing Lin Feng; Email: fxl@bjmu.edu.cn

Citation: Zhao B, Cheng Z, Feng XL. Understanding heterogeneous drug procurement behaviour of healthcare institutions under pooled procurement: evidence from China. Int J Health Policy Manag. 2026;15:9372. doi:10.34172/ijhpm.9372

Supplementary file 2

List of contents

Figure S1. Estimated Trends from ITS on Bid-Winning Product Procurement by Pre-Policy Bid-Winning Share (Five-Level Stratification) 3

Figure S2. Sensitivity Analyses: Estimated Trends from ITS on Bid-Winning Product Procurement by Pre-Policy Bid-Winning Share (Three-Level Stratification) 4

Figure S3. Sensitivity Analyses: Estimated Trends from ITS on Bid-Winning Product Procurement by Pre-Policy Bid-Winning Share (Median Split)...4

Figure S4. Sensitivity Analyses: Estimated Trends from ITS on Bid-Winning Product Procurement by Pre-Policy Bid-Winning Share (Mean Split)...4

Figure S5. Estimated Trends from ITS on Alternative Drug Procurement by Pre-Policy NVBP Share (Five-Level Stratification)...5

Figure S6. ITS-Based Forest Plot of Post-Policy Changes in Alternative Drug Procurement Volume by Pre-Policy NVBP Share (Stratified in 0.05 and 0.01 increments)...6

Figure S7. Sensitivity Analyses: Estimated Trends from ITS on Alternative Drug Procurement by Pre-Policy NVBP Share (Three-Level Stratification)..6

Figure S8. Sensitivity Analyses: Estimated Trends from ITS on Alternative Drug Procurement by Pre-Policy NVBP Share (Median Split)..7

Figure S9. Sensitivity Analyses: Estimated Trends from ITS on Alternative Drug Procurement by Pre-Policy NVBP Share (Mean Split)..7

Figure S10. ITS-Based Forest Plot of Post-Policy Changes in Alternative Drug Procurement Volume by Pre-Policy NVBP Share for the three alternative drug categories...8

Figures

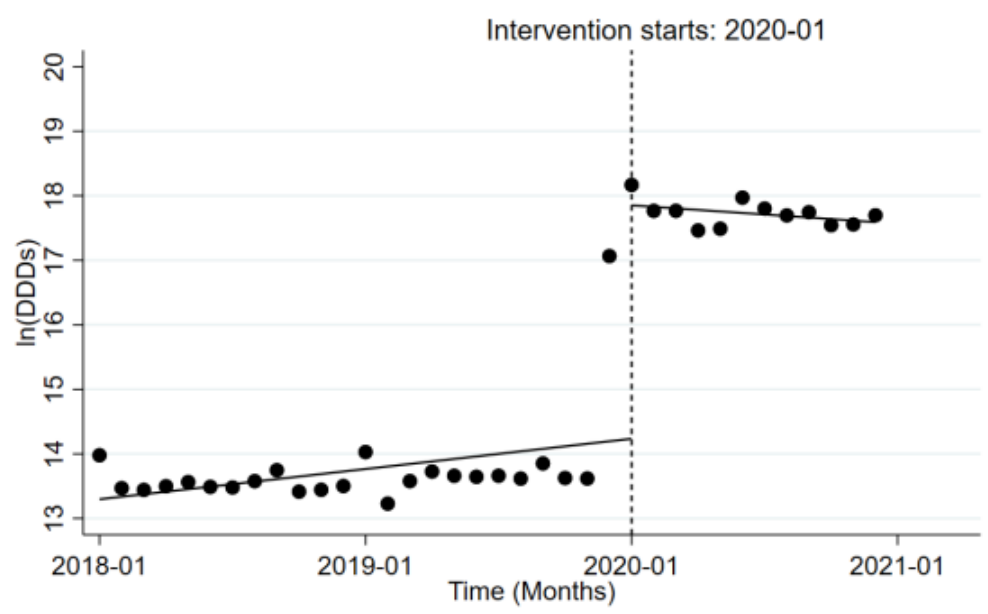

(a) Very Low Share Subgroup

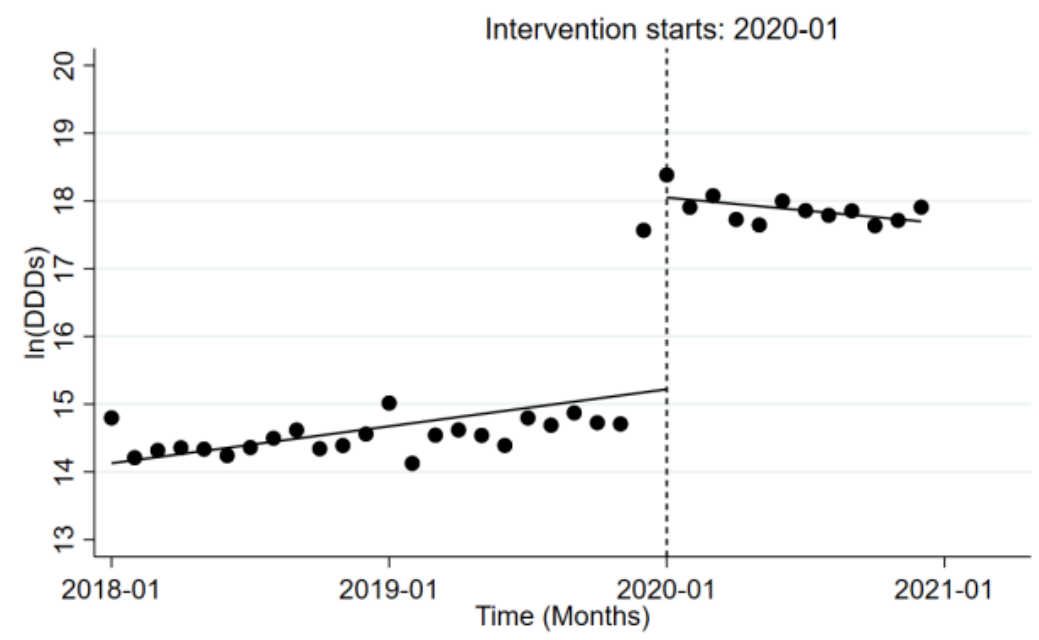

(b) Low Share Subgroup

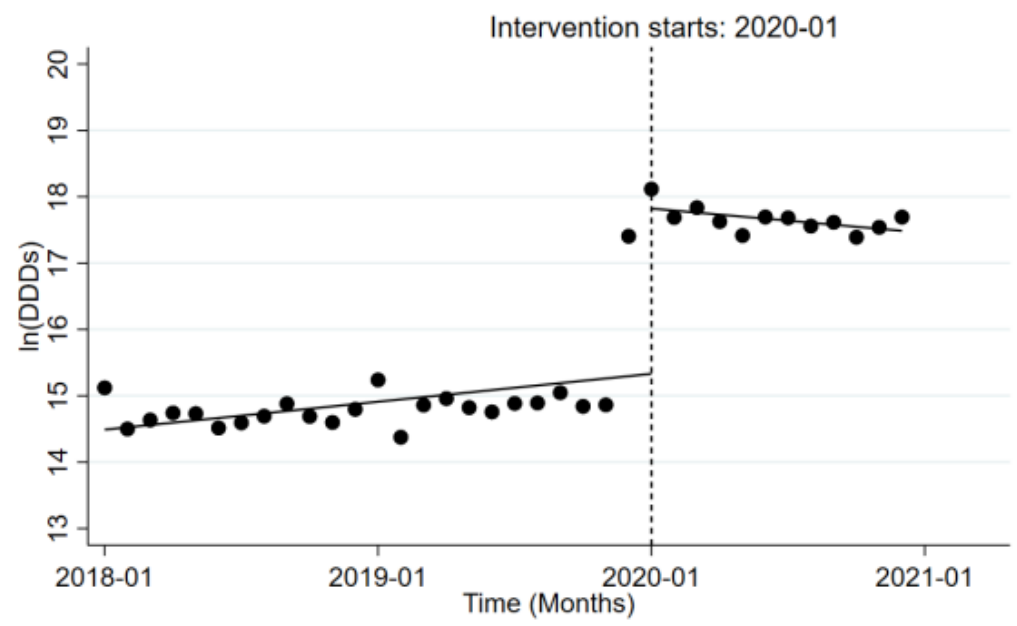

(c) Moderate Share Subgroup

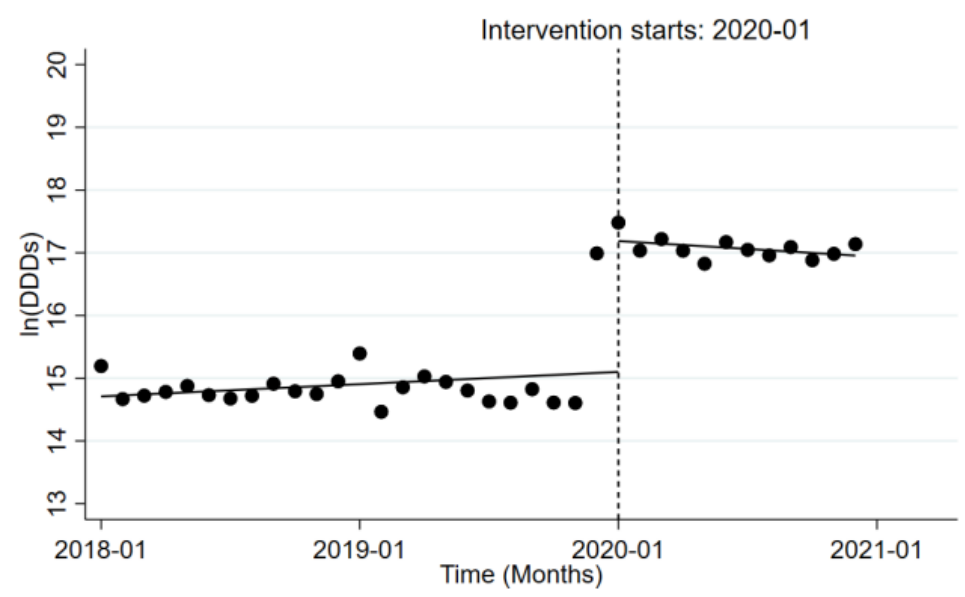

(d) High Share Subgroup

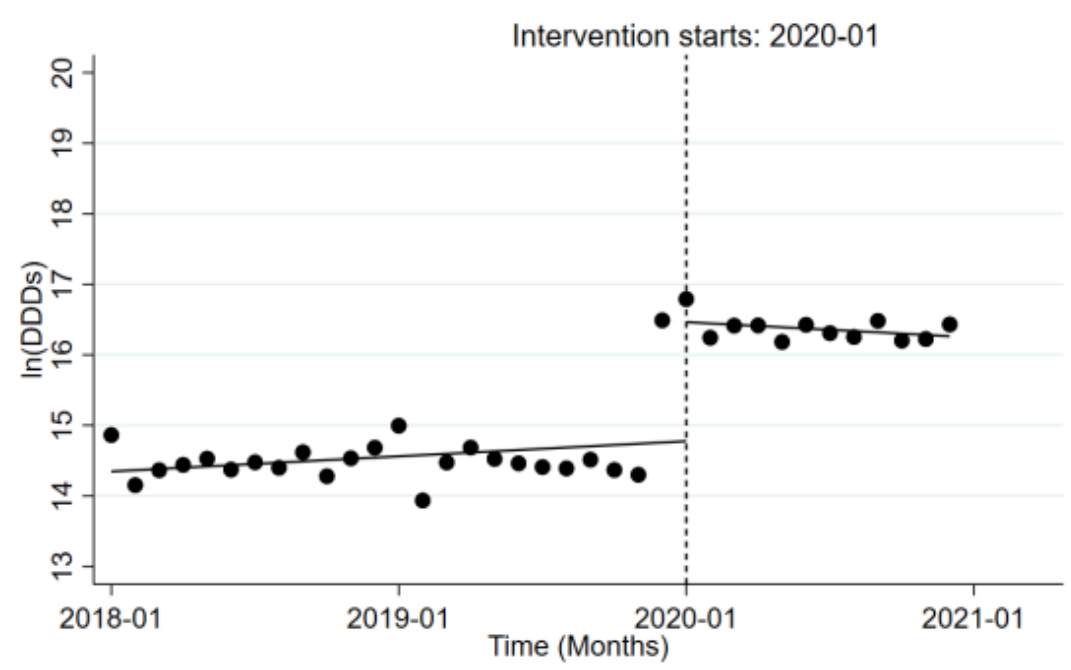

(e) Very High Share Subgroup

Figure S1. Estimated Trends from ITS on Bid-Winning Product Procurement by Pre-Policy Bid-Winning Share (Five-Level Stratification)

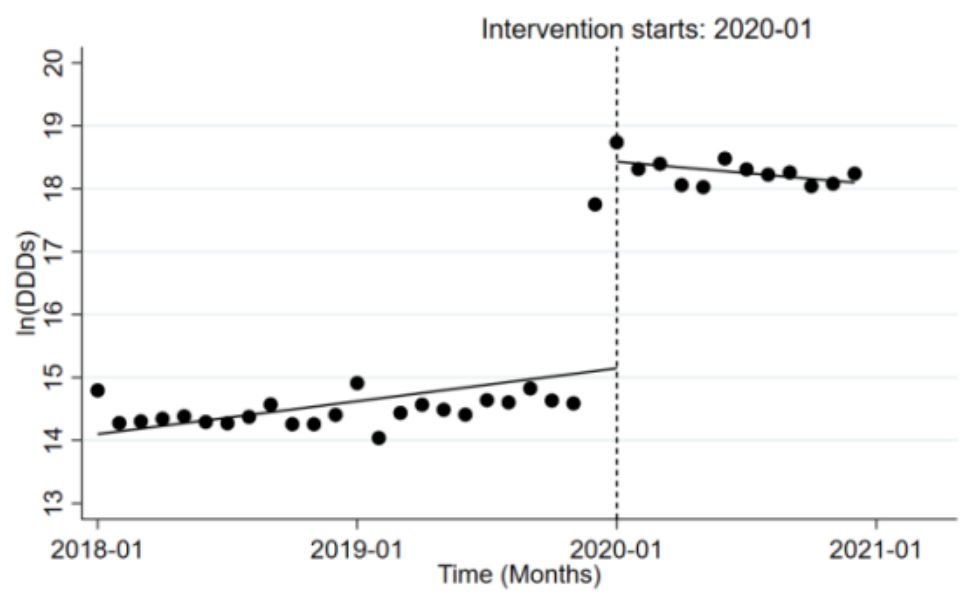

(a)Low Share Subgroup

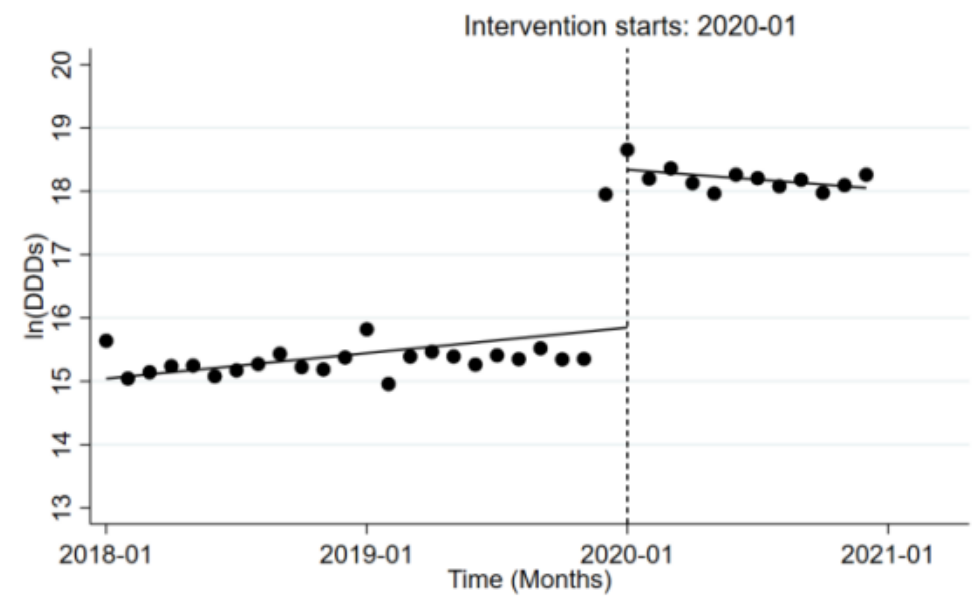

(b)Moderate Share Subgroup

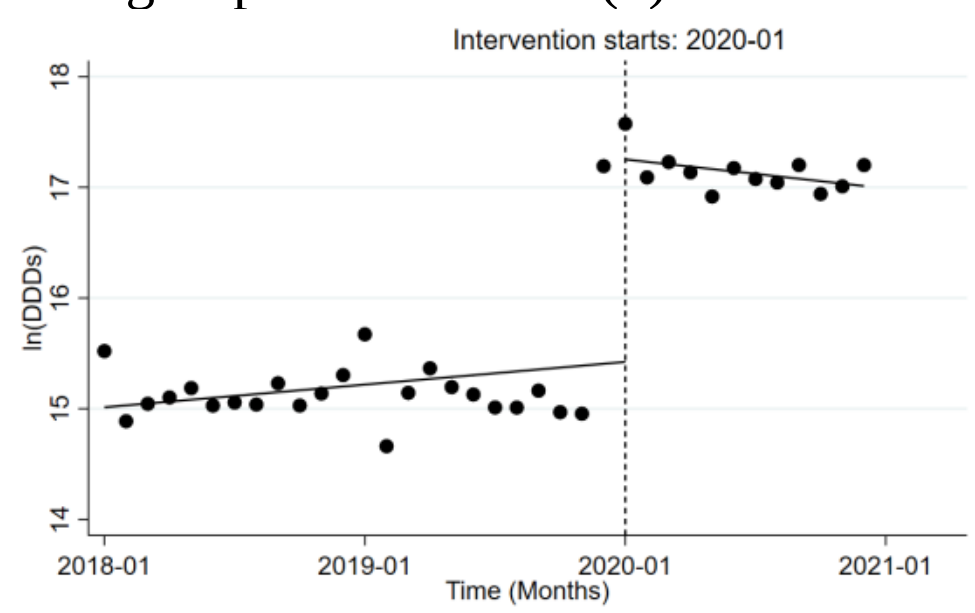

(c)High Share Subgroup

Figure S2. Sensitivity Analyses: Estimated Trends from ITS on Bid-Winning Product Procurement by Pre-Policy Bid-Winning Share (Three-Level Stratification)

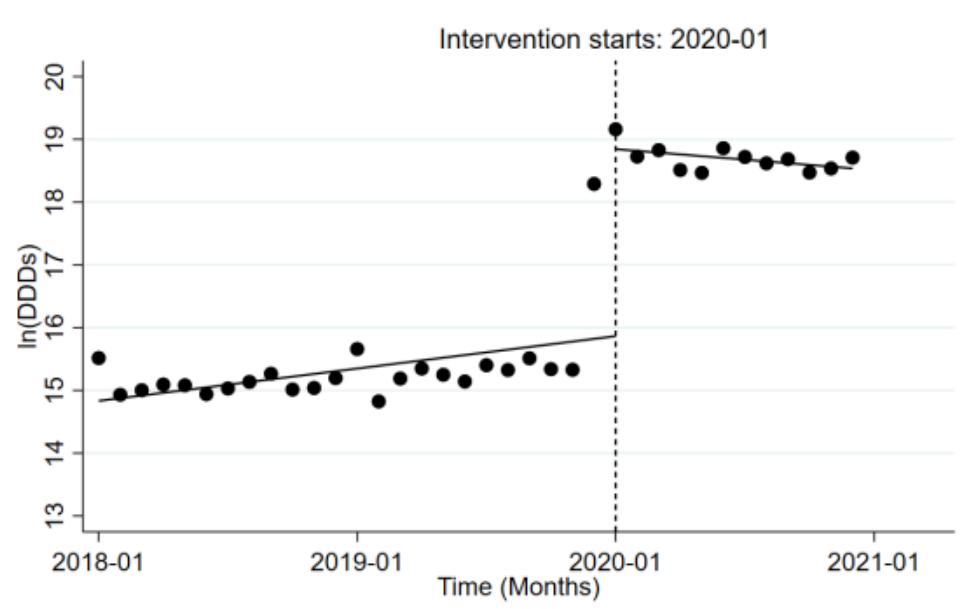

(a)Low Share Subgroup

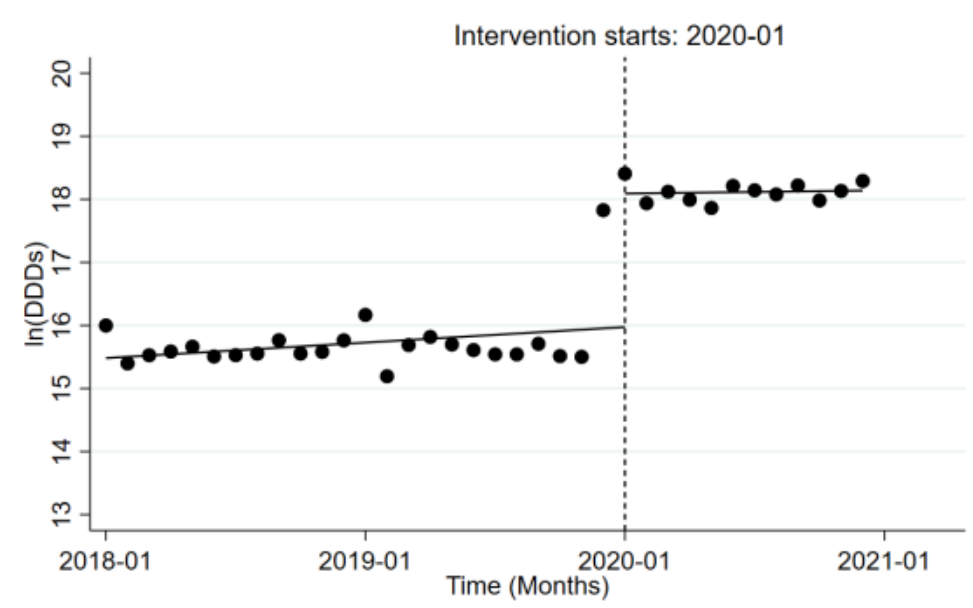

(b)High Share Subgroup

Figure S3. Sensitivity Analyses: Estimated Trends from ITS on Bid-Winning Product Procurement by Pre-Policy Bid-Winning Share (Median Split)

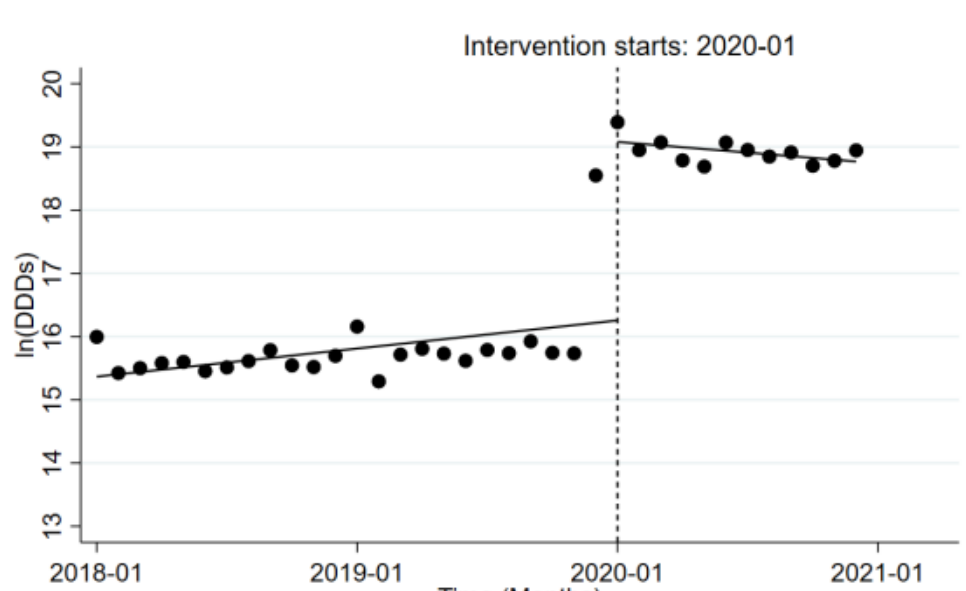

(a)Low Share Subgroup

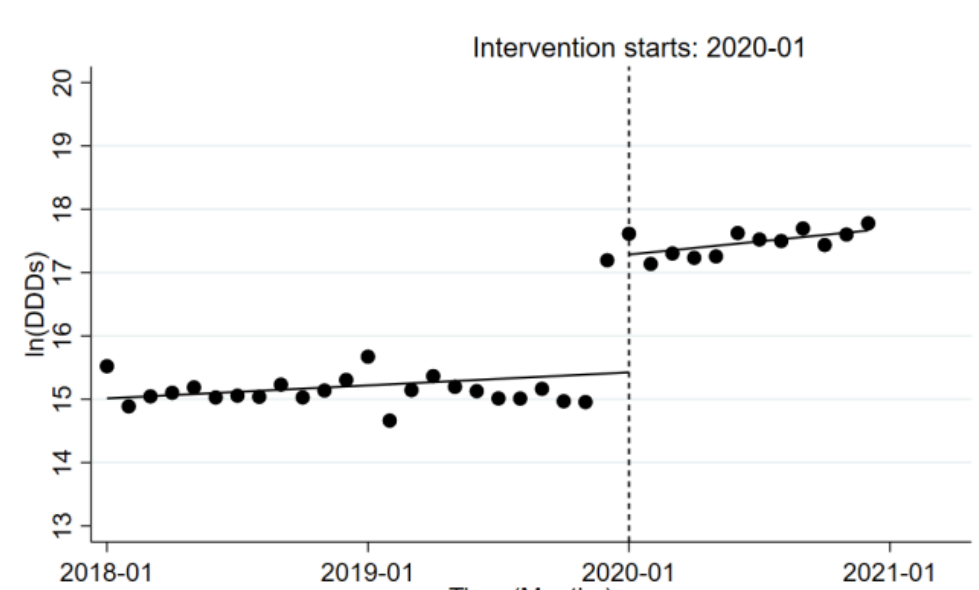

(b)High Share Subgroup

Figure S4. Sensitivity Analyses: Estimated Trends from ITS on Bid-Winning Product Procurement by Pre-Policy Bid-Winning Share (Mean Split)

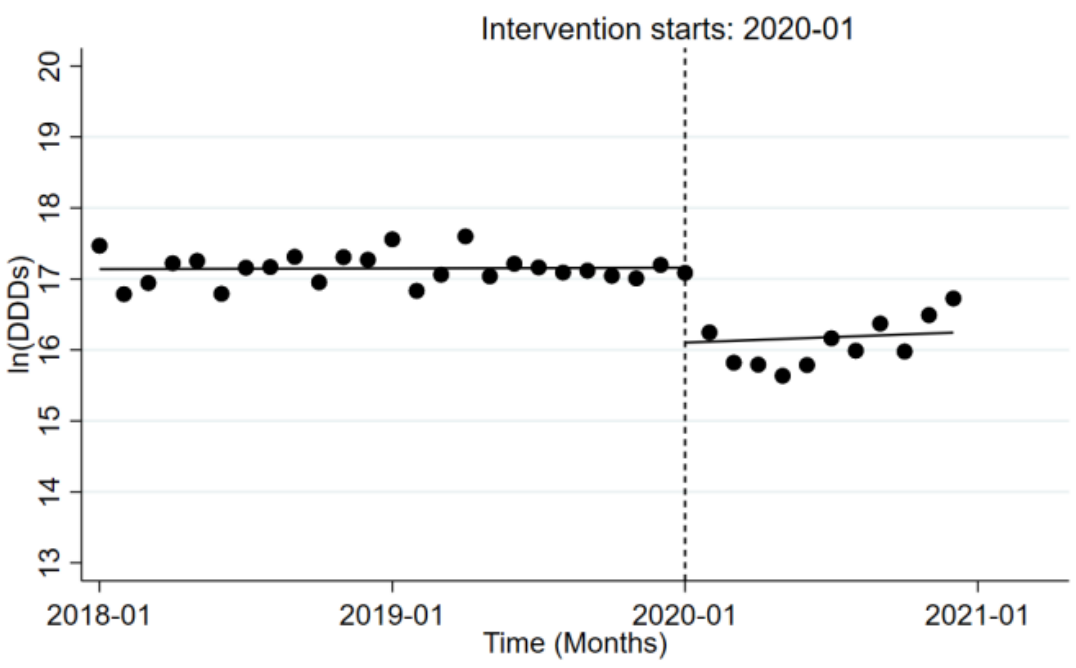

(a)Very Low Share Subgroup

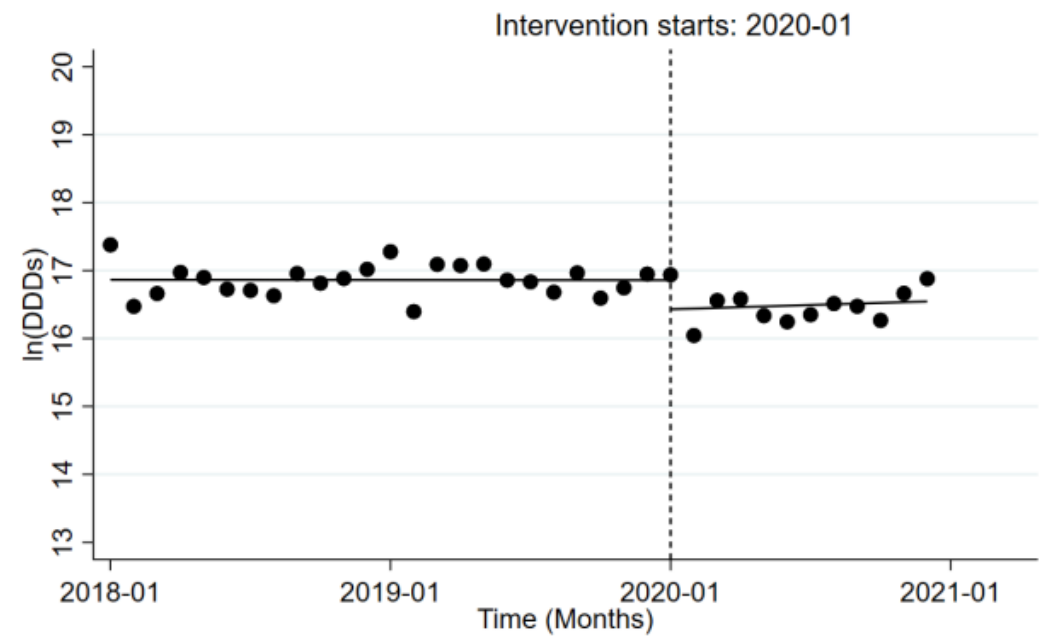

(b)Low Share Subgroup

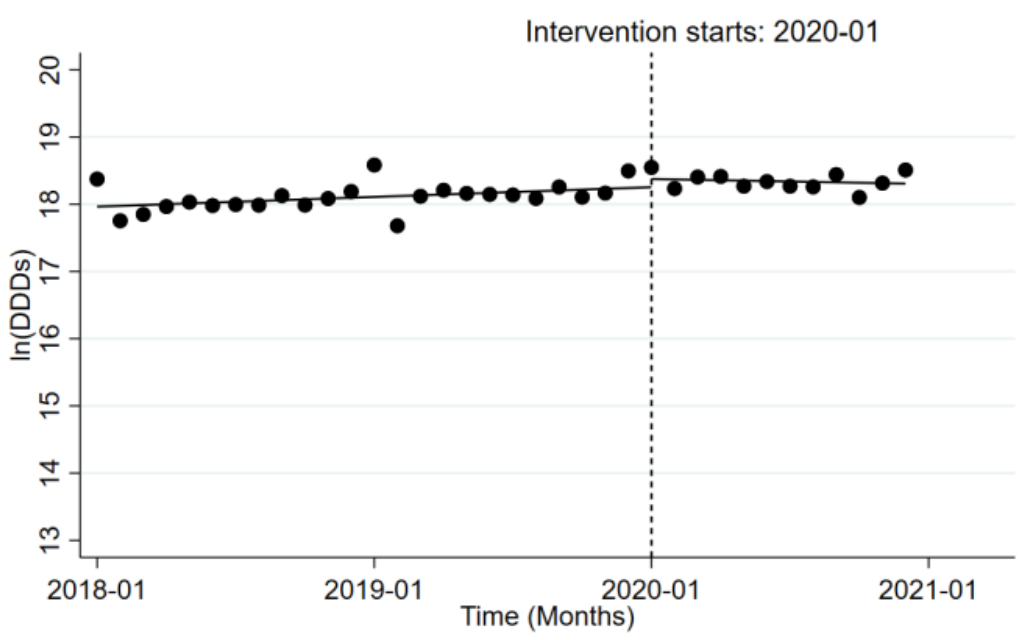

(c)Moderate Share Subgroup

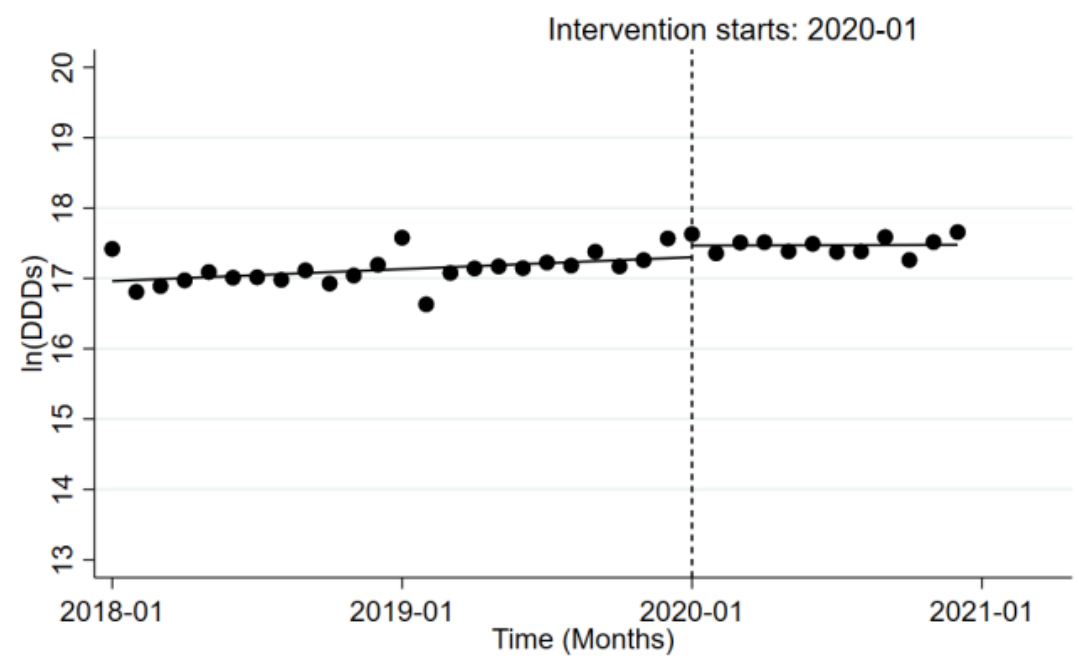

(d)High Share Subgroup

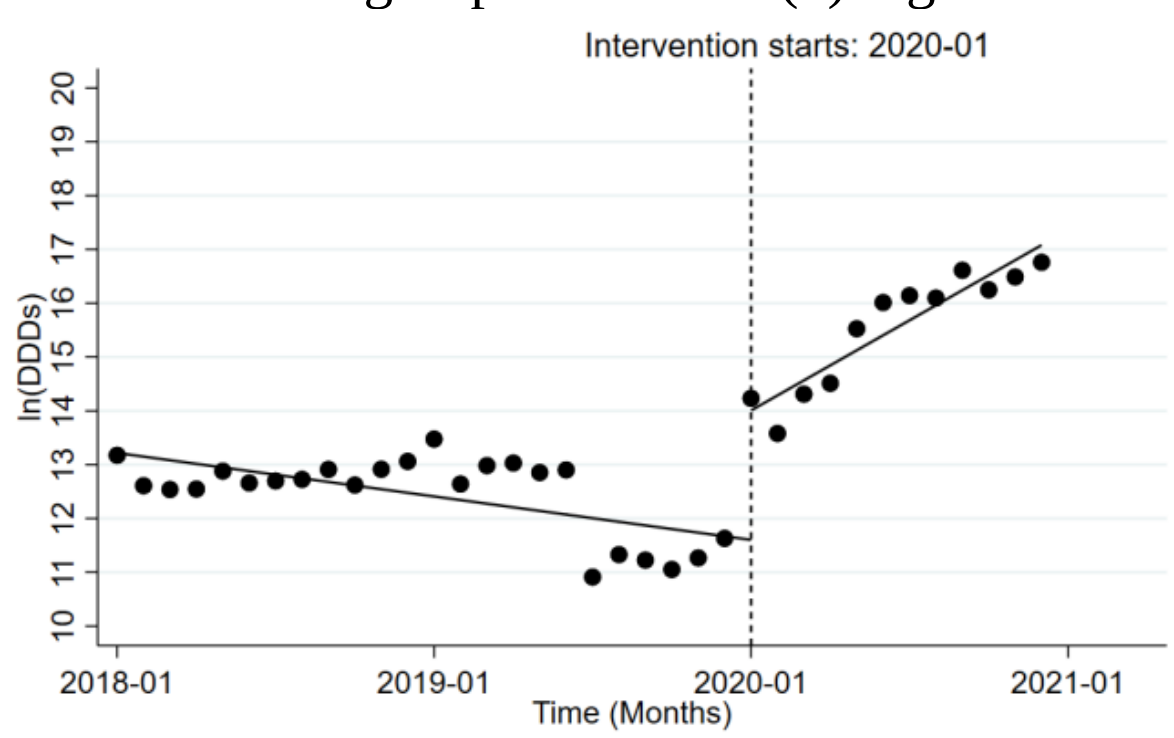

(e)Very High Share Subgroup

Figure S5. Estimated Trends from ITS on Alternative Drug Procurement by Pre-Policy NVBP Share (Five-Level Stratification)

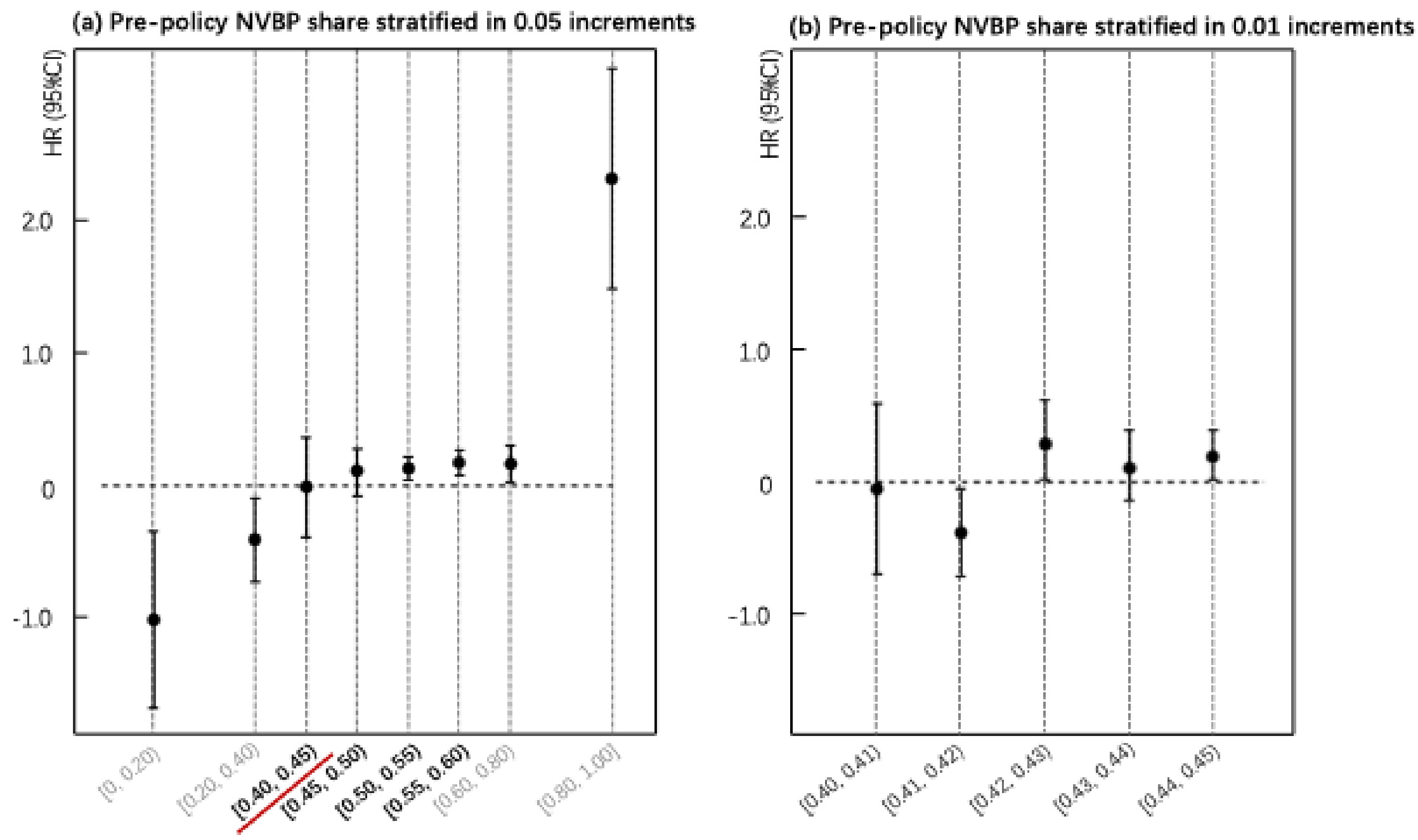

Figure S6. ITS-Based Forest Plot of Post-Policy Changes in Alternative Drug Procurement Volume by Pre-Policy NVBP Share (Stratified in 0.05 and 0.01 increments)
Note: The figure only displays estimated post-policy level changes (β_2) and their 95% confidence intervals from ITS regressions. Trend changes (β_3) are not shown, as they were consistently insignificant.

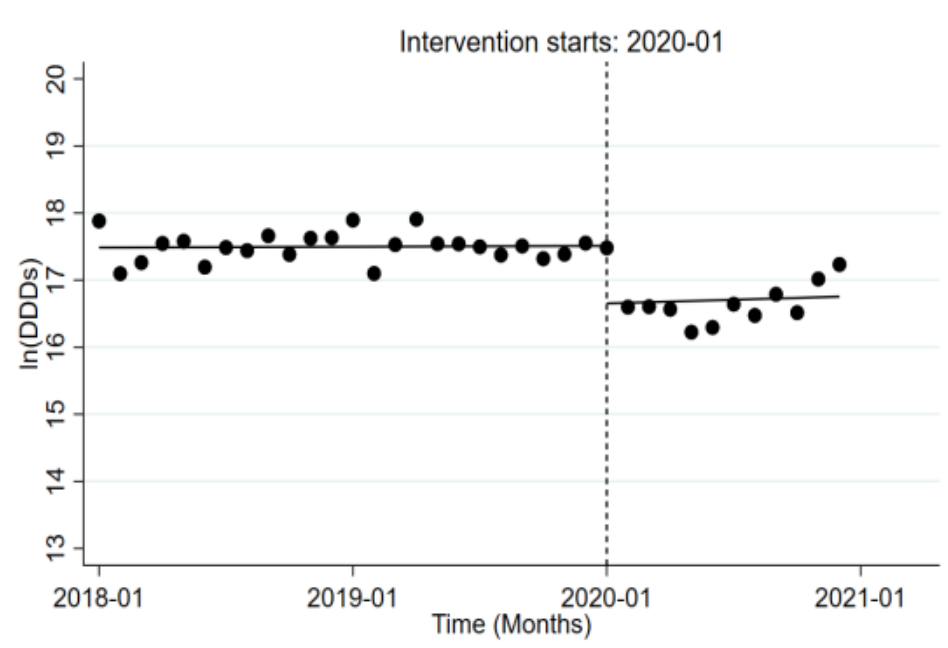

(a)Low Share Subgroup

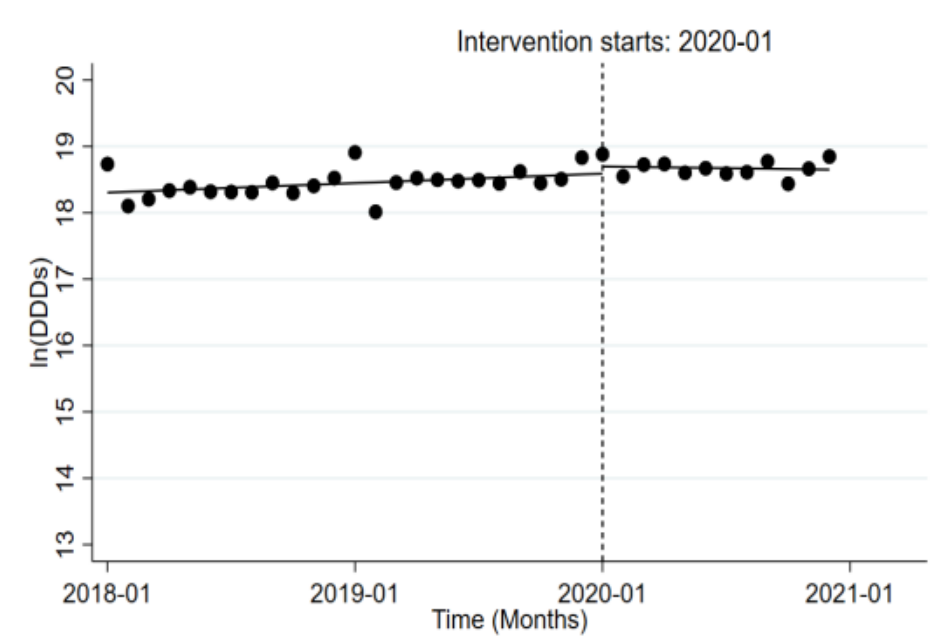

(b)Moderate Share Subgroup

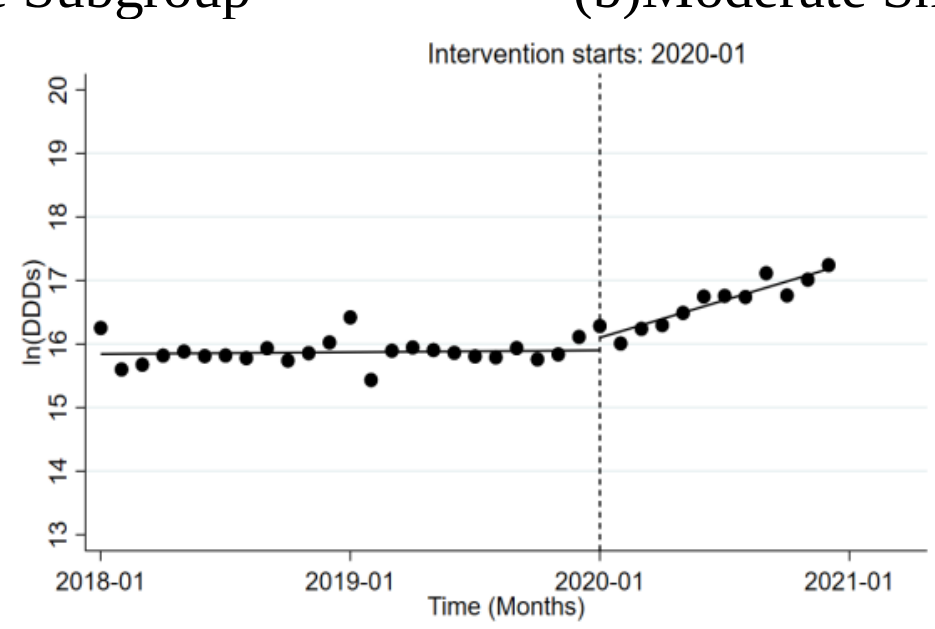

(c)High Share Subgroup

Figure S7. Sensitivity Analyses: Estimated Trends from ITS on Alternative Drug Procurement by Pre-Policy NVBP Share (Three-Level Stratification)

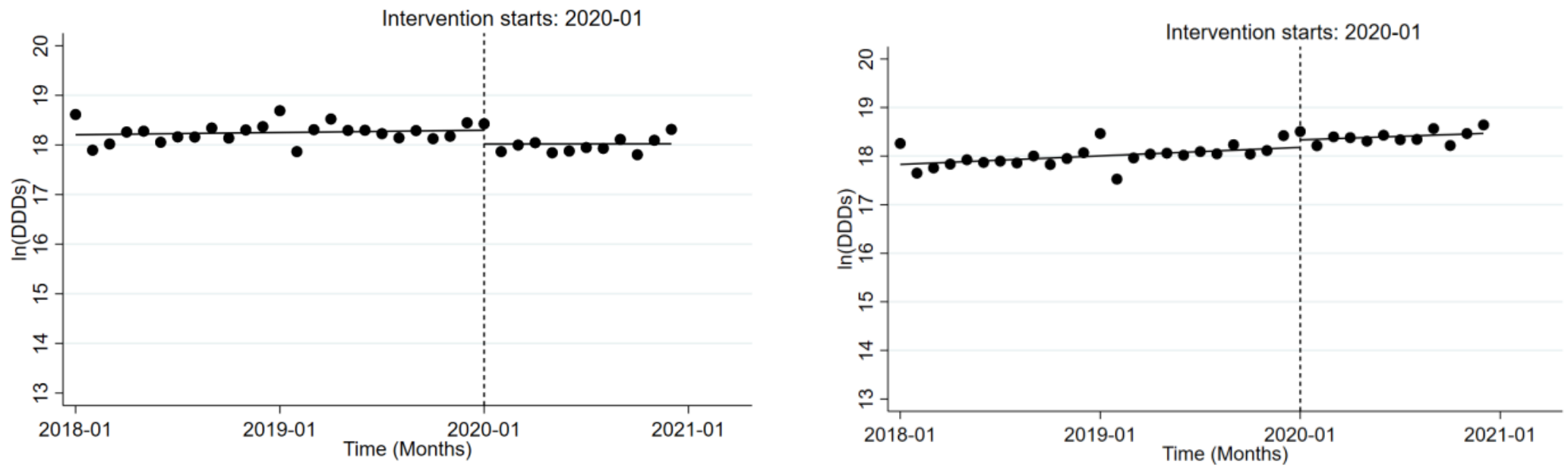

(a)Low Share Subgroup (b)High Share Subgroup

Figure S8. Sensitivity Analyses: Estimated Trends from ITS on Alternative Drug Procurement by Pre-Policy NVBP Share (Median Split)

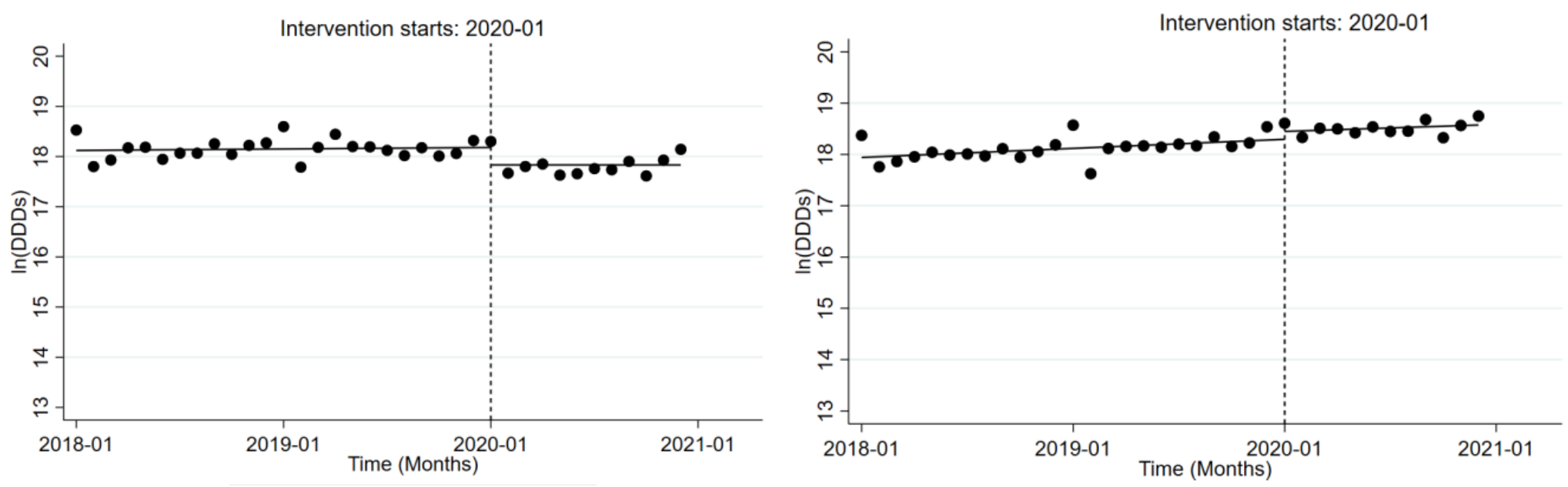

(a)Low Share Subgroup (b)High Share Subgroup

Figure S9. Sensitivity Analyses: Estimated Trends from ITS on Alternative Drug Procurement by Pre-Policy NVBP Share (Mean Split)

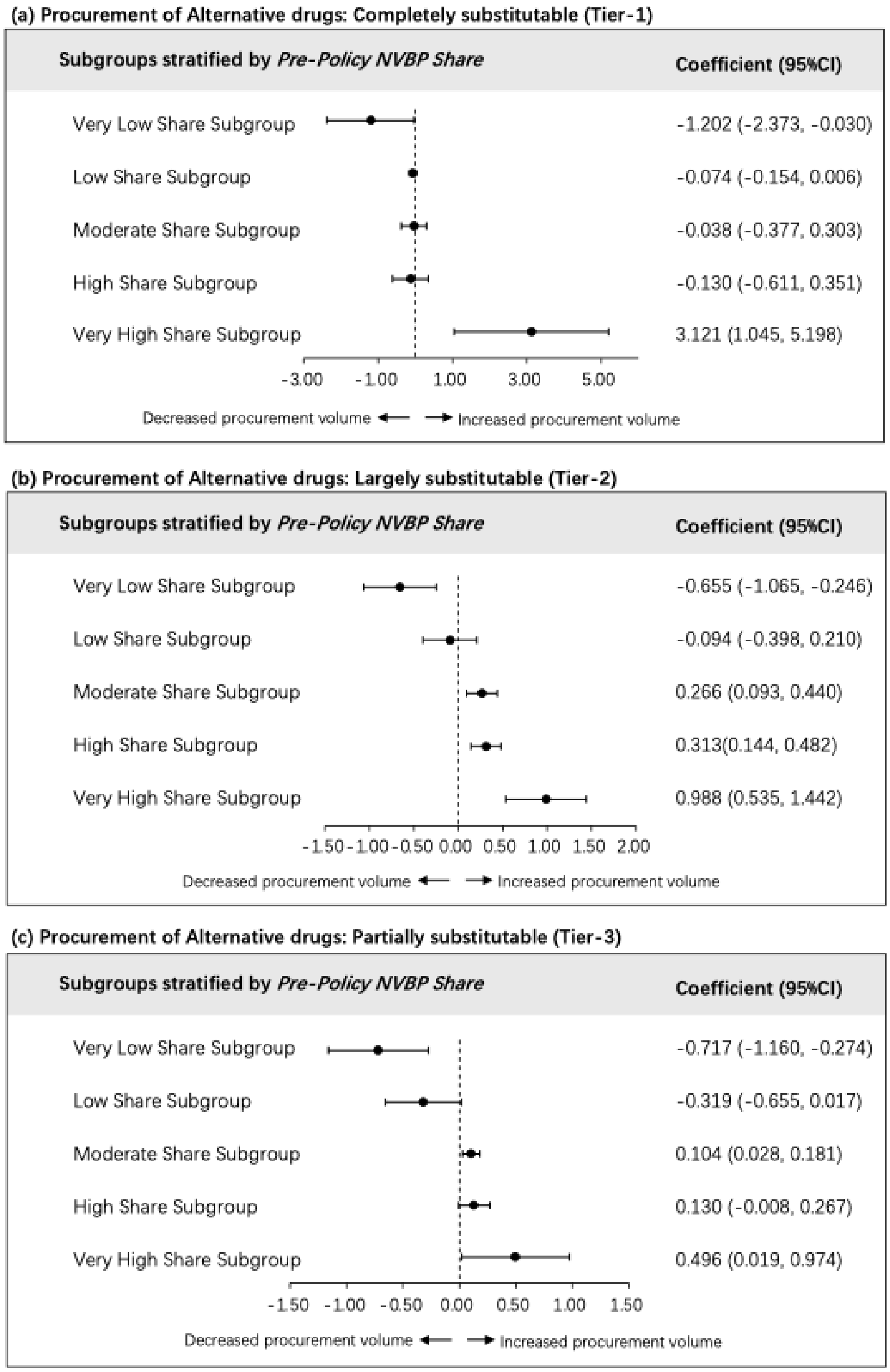

Figure S10. ITS-Based Forest Plot of Post-Policy Changes in Alternative Drug Procurement Volume by Pre-Policy NVBP Share for the three alternative drug categories

Note: The figure only displays estimated post-policy level changes (β_2) and their 95% confidence intervals from ITS regressions. Trend changes (β_3) are not shown, as they were consistently insignificant.
