# Supplementary material for: Understanding Heterogeneous Drug Procurement Behaviour of Healthcare Institutions Under Pooled Procurement: Evidence From China
Source: Int J Health Policy Manag. 2026 Jun 7;15:9372. doi: 10.34172/ijhpm.9372 (PMC13729802; doi:10.34172/ijhpm.9372)
Supplement: Supplementary file 3 [file ijhpm-15-9372-s003.pdf]

Article title: Understanding Heterogeneous Drug Procurement Behaviour of Healthcare Institutions Under Pooled Procurement: Evidence From China

Journal name: International Journal of Health Policy and Management (IJHPM)

Authors' information: Boya Zhao, Zhao Cheng, Xing Lin Feng*

School of Public Health, Peking University, Beijing, China.

***Correspondence to:** Xing Lin Feng; Email: fxl@bjmu.edu.cn

Citation: Zhao B, Cheng Z, Feng XL. Understanding heterogeneous drug procurement behaviour of healthcare institutions under pooled procurement: evidence from China. Int J Health Policy Manag. 2026;15:9372. doi:10.34172/ijhpm.9372

Supplementary file 3

List of contents

Table S11. Descriptive Statistics of Institutional Subgroups by Pre-Policy NVBP Share......................... 1

Table S12. ITS Regression Results for Procurement Volume of Alternative Medicine by Pre-Policy NVBP Share (Five-Level Stratification) 1

Table S13. ITS Regression Results for Procurement Expenditure of Alternative Medicine by Pre-Policy NVBP Share (Five-Level Stratification)... 2

Table S14. Sensitivity Analyses: ITS Regression Results on Alternative Medicine Procurement by Pre-Policy NVBP Share (Three-Level Stratification) 2

Table S15. Sensitivity Analyses: ITS Regression Results on Alternative Medicine Procurement by Pre-Policy NVBP Share (Median split) 3

Table S16. Sensitivity Analyses: ITS Regression Results on Alternative Medicine Procurement by Pre-Policy NVBP Share (Mean split) 3

Table S17. ITS Regression Results for Procurement Volume (DDDs) of NVBP-Covered Drugs at Fictional Time Points for Placebo Test... 3

Table S18. ITS Regression Results for Procurement Volume (DDDs) of Bid-winning Products at Fictional Time Points for Placebo Test... 4

Table S19. ITS Regression Results for Procurement Volume (DDDs) of Non-winning Products at Fictional Time Points for Placebo Test... 4

Table S20. ITS Regression Results for Procurement Volume (DDDs) of Alternative Drugs at Fictional Time Points for Placebo Test... 4

Table S11. Descriptive Statistics of Institutional Subgroups by Pre-Policy NVBP Share

**Subgroup**	Obs	Mean	Std. Dev.	Min	Max
**Binary Stratification (Median Split)**					
Low share subgroup	63,018	0.3751	0.1135	0.0010	0.5260
High share subgroup	63,015	0.6386	0.0823	0.5261	0.9994
**Binary Stratification (by Median)**					
Low share subgroup	57,779	0.3623	0.1098	0.0010	0.5068

High share subgroup	68,254	0.6292	0.0855	0.5069	0.9994
**Three-level Stratification**					
Low share subgroup	19,917	0.2350	0.0720	0.0010	0.3300
Moderate share subgroup	84,938	0.5139	0.0889	0.3301	0.6598
High share subgroup	21,178	0.7341	0.0613	0.6601	0.9994
**Five-level Stratification**					
Very Low share subgroup	5,718	0.1412	0.0483	0.0010	0.1999
Low share subgroup	25,897	0.3142	0.0548	0.2000	0.4000
Moderate share subgroup	55,768	0.5099	0.0563	0.4000	0.5999
High share subgroup	35,764	0.6722	0.0537	0.6000	0.8000
Very High share subgroup	2,886	0.8517	0.0440	0.8000	0.9994

Table S12. ITS Regression Results for Procurement Volume of Alternative Drug by Pre-Policy NVBP Share (Five-Level Stratification)

	Very Low Share Subgroup	Low Share Subgroup	Moderate Share Subgroup	High Share Subgroup	Very High Share Subgroup
T_t	0.000792	-0.000251	0.0121^{**}	0.0141^{**}	-0.0673^{***}
	(0.00625)	(0.00687)	(0.00521)	(0.00557)	(0.0204)
X_t	-1.055^{***}	-0.428^{***}	0.122^{*}	0.166^{**}	2.405^{***}
	(0.351)	(0.166)	(0.0714)	(0.0738)	(0.439)
X_tT_t	0.0120	0.0107	-0.0183	-0.0132	0.347^{***}
	(0.0481)	(0.0258)	(0.0132)	(0.0128)	(0.0409)
Constant	17.14^{***}	16.86^{***}	17.96^{***}	16.96^{***}	13.22^{***}
	(0.0987)	(0.102)	(0.0747)	(0.0789)	(0.215)

Table S13. ITS Regression Results for Procurement Expenditure of Alternative Medicine by Pre-Policy NVBP Share (Five-Level Stratification)

	Very Low Share Subgroup	Low Share Subgroup	Moderate Share Subgroup	High Share Subgroup	Very High Share Subgroup
T_t	0.00401	0.00664	0.0159^{***}	0.0309^{***}	-0.0652^{***}
	(0.00622)	(0.00573)	(0.00543)	(0.00573)	(0.0124)
X_t	-0.603^{**}	-0.266^{**}	0.0292	-0.0162	2.135^{***}
	(0.268)	(0.126)	(0.113)	(0.135)	(0.337)
X_tT_t	0.0297	0.0133	-0.0251	-0.0479^{***}	0.394^{***}
	(0.0354)	(0.0191)	(0.0160)	(0.0169)	(0.0392)
Constant	16.38^{***}	17.21^{***}	18.94^{***}	18.09^{***}	14.13^{***}
	(0.0950)	(0.0843)	(0.0803)	(0.0874)	(0.134)

Table S14. Sensitivity Analyses: ITS Regression Results on Alternative Medicine Procurement by Pre-Policy NVBP Share (Three-Level Stratification)

	Low Share Subgroup	Moderate Share Subgroup	High Share Subgroup
T_t	0.00105	0.0119^{***}	0.00239
	(0.00688)	(0.00438)	(0.00595)
X_t	-0.858^{***}	0.107^{*}	0.200^{*}
	(0.294)	(0.0581)	(0.113)
X_tT_t	0.00811	-0.0161	0.0959^{***}
	(0.0417)	(0.0110)	(0.0142)
Constant	17.48^{***}	18.30^{***}	15.84^{***}
	(0.107)	(0.0634)	(0.0894)

Table S15. Sensitivity Analyses: ITS Regression Results on Alternative Medicine Procurement by Pre-Policy NVBP Share (Median split)

	Low Share Subgroup	High Share Subgroup
T_t	0.00376	0.0146***
	(0.00636)	(0.00518)
X_t	-0.277*	0.154**
	(0.163)	(0.0693)
X_tT_t	-0.00335	-0.00256
	(0.0230)	(0.0125)
Constant	18.21***	17.83***
	(0.0961)	(0.0743)

Table S16. Sensitivity Analyses: ITS Regression Results on Alternative Medicine Procurement by Pre-Policy NVBP Share (Mean split)

	Low Share Subgroup	High Share Subgroup
T_t	0.00246	0.0147***
	(0.00641)	(0.00519)
X_t	-0.347*	0.153**
	(0.180)	(0.0680)
X_tT_t	-0.00257	-0.00338
	(0.0254)	(0.0123)
Constant	18.12***	17.94***
	(0.0971)	(0.0745)

Table S17. ITS Regression Results for Procurement Volume (DDDs) of NVBP-Covered Drugs at Fictional Time Points for Placebo Test

	(1)	(2)	(3)	(4)	(5)
	2018-06	2018-09	2018-12	2019-03	2019-06
Constant	18.72***	18.68***	18.65***	18.64***	18.62***
	(0.197)	(0.157)	(0.130)	(0.129)	(0.0968)
T_t	-0.0317	-0.0120	0.00179	0.00673	0.0107
	(0.0642)	(0.0302)	(0.0186)	(0.0219)	(0.0116)
X_t	0.0623	0.0991	0.0681	-0.00243	-0.0911
	(0.162)	(0.138)	(0.187)	(0.209)	(0.140)
X_tT_t	0.0497	0.0288	0.0151	0.0216	0.0447
	(0.0647)	(0.0326)	(0.0297)	(0.0319)	(0.0397)

Table S18. ITS Regression Results for Procurement Volume (DDDs) of Bid-winning Products at Fictional Time Points for Placebo Test

	(1)	(2)	(3)	(4)	(5)
	2018-06	2018-09	2018-12	2019-03	2019-06
Constant	16.22***	16.17***	16.12***	16.09***	16.07***
	(0.213)	(0.174)	(0.145)	(0.143)	(0.113)
T_t	-0.0567	-0.0287	-0.00959	0.00145	0.00700
	(0.0695)	(0.0344)	(0.0211)	(0.0232)	(0.0128)
X_t	-0.0938	-0.0478	-0.115	-0.286	-0.503
	(0.275)	(0.294)	(0.354)	(0.396)	(0.389)
X_tT_t	0.105	0.0868	0.0890	0.135	0.276
	(0.0792)	(0.0630)	(0.0806)	(0.117)	(0.191)

Table S19. ITS Regression Results for Procurement Volume (DDDs) of Non-winning Products at Fictional Time Points for Placebo Test

	(1)	(2)	(3)	(4)	(5)
	2018-06	2018-09	2018-12	2019-03	2019-06
Constant	18.46***	18.42***	18.38***	18.38***	18.36***
	(0.210)	(0.169)	(0.140)	(0.139)	(0.104)
T_t	-0.0339	-0.00942	0.00377	0.00765	0.0122
	(0.0694)	(0.0327)	(0.0199)	(0.0237)	(0.0126)
X_t	0.132	0.159	0.145	0.118	0.0889
	(0.176)	(0.143)	(0.189)	(0.221)	(0.157)
X_tT_t	0.0449	0.0155	-0.00425	-0.0124	-0.0348
	(0.0696)	(0.0340)	(0.0279)	(0.0271)	(0.0255)

Table S20. ITS Regression Results for Procurement Volume (DDDs) of Alternative Drugs at Fictional Time Points for Placebo Test

	(1)	(2)	(3)	(4)	(5)
	2018-06	2018-09	2018-12	2019-03	2019-06
Constant	18.83***	18.80***	18.75***	18.74***	18.72***
	(0.254)	(0.201)	(0.167)	(0.155)	(0.122)
T_t	-0.0381	-0.0175	-0.00000994	0.00602	0.0101
	(0.0824)	(0.0385)	(0.0239)	(0.0236)	(0.0134)
X_t	0.105	0.169	0.120	0.0388	-0.0866
	(0.199)	(0.150)	(0.188)	(0.208)	(0.139)
X_tT_t	0.0490	0.0236	0.00232	0.000859	0.0169
	(0.0826)	(0.0396)	(0.0305)	(0.0279)	(0.0260)
